# Impact of gulf war toxic exposures after mild traumatic brain injury

**DOI:** 10.1186/s40478-022-01449-x

**Published:** 2022-10-18

**Authors:** Scott Ferguson, Robyn McCartan, Mackenzie Browning, Coral Hahn-Townsend, Arissa Gratkowski, Alexander Morin, Laila Abdullah, Ghania Ait-Ghezala, Joseph Ojo, Kimberly Sullivan, Michael Mullan, Fiona Crawford, Benoit Mouzon

**Affiliations:** 1 Roskamp Institute, 2040 Whitfield Ave, Sarasota, FL 34243 USA; 2 James A. Haley Veterans’ Hospital, Tampa, FL USA; 3 Department of Environmental Health, School of Public Health, Boston University, 715 Albany St. T4W, Boston, MA 02118 USA

**Keywords:** Traumatic brain injury, Gulf war illness, Toxic exposures, Neuroinflammation, Chemical exposure, White matter, Tau, Cognitive dysfunction, Depression, Concussion

## Abstract

Chemical and pharmaceutical exposures have been associated with the development of Gulf War Illness (GWI), but how these factors interact with the pathophysiology of traumatic brain injury (TBI) remains an area of study that has received little attention thus far. We studied the effects of pyridostigmine bromide (an anti-nerve agent) and permethrin (a pesticide) exposure in a mouse model of repetitive mild TBI (r-mTBI), with 5 impacts over a 9-day period, followed by Gulf War (GW) toxicant exposure for 10 days beginning 30 days after the last head injury. We then assessed the chronic behavioral and pathological sequelae 5 months after GW agent exposure. We observed that r-mTBI and GWI cumulatively affect the spatial memory of mice in the Barnes maze and result in a shift of search strategies employed by r-mTBI/GW exposed mice. GW exposure also produced anxiety-like behavior in sham animals, but r-mTBI produced disinhibition in both the vehicle and GW treated mice. Pathologically, GW exposure worsened r-mTBI dependent axonal degeneration and neuroinflammation, increased oligodendrocyte cell counts, and increased r-mTBI dependent phosphorylated tau, which was found to colocalize with oligodendrocytes in the corpus callosum. These results suggest that GW exposures may worsen TBI-related deficits. Veterans with a history of both GW chemical exposures as well as TBI may be at higher risk for worse symptoms and outcomes. Subsequent exposure to various toxic substances can influence the chronic nature of mTBI and should be considered as an etiological factor influencing mTBI recovery.

**Supplementary Information:**

The online version contains supplementary material available at 10.1186/s40478-022-01449-x.

## Introduction

Gulf War Illness (GWI) is a chronic multisystem illness that affects approximately one third of all Gulf War (GW) veterans [1, 2]. Symptoms of GWI include chronic pain, fatigue, cognitive decrements, and gastrointestinal complaints [66]. The causes of GWI remain to be fully elucidated, but exposure to chemical and other environmental stressors in the GW theatre are thought to be contributing factors [25, 49]. These exposures ranged from pharmaceuticals such as pyridostigmine bromide (PB) which was used as a prophylactic against nerve gas exposure, excessive exposures to pesticides and insecticides, deployment and combat stress under intense heat, depleted uranium, heat and smoke from oil-well fires, and industrial solvents and gases [66].

In addition to being exposed to these environmental hazards, the military population is particularly vulnerable to traumatic brain injury (TBI). Since 2000, the Defense and Veterans Brain Injury Center (DVBIC) has reported nearly 350,000 TBI incidents in the U.S. military with 59% of those affected reporting more than one TBI [22] with the majority of them being concussion or mild TBI (mTBI) [13]. While TBI is not considered a hallmark of the Gulf War (GW), a study examining a cohort of GW veterans showed that 36% of GW veterans reported sustaining a mTBI during deployment. That same study demonstrated that veterans exposed to GW toxicants and mTBI were more likely to develop GWI than veterans only exposed to GW toxicants [32]. Furthermore, veterans with a history of TBI and GW toxicant exposure have reported increased rates of chronic multisystem illness and a poorer health related quality of life in comparison to veterans only exposed to GW toxicants [70]. When exposed to three or more mTBIs, the risk of chronic health symptoms and diagnosis of GWI was significantly increased [69].

Research from the past decade has drawn much attention to the long-term pathological consequences of TBI [27, 28, 59]. For example, autopsy-derived tissue from athletes indicated a persistent neuroinflammation many years after r-mTBI [24, 27]. Postmortem tissue of GW veterans is currently lacking and the pathological hallmarks of GWI, if any, remain unknown. In the absence of these neuropathological assessments, we rely on neuropathological findings in preclinical models of GWI [55] and in vivo imaging studies in GW veterans [7, 14, 15]. Therefore, we hypothesized that a history of multiple mTBIs before deployment may exacerbate the central nervous system inflammatory response and produce a persistent “priming” of the immune system, continuing to generate heightened responses to subsequent chemical and/or environmental stressors such as those to which GW veterans were exposed in theatre.

Despite the importance of polytrauma and secondary insults in TBI, only a few preclinical studies have evaluated the presence of concurrent secondary insult as a determinant outcome after TBI [10, 57], and to the best of our knowledge none on the delayed chronic effects of mTBI. Considering that the majority of mTBIs occur during basic training for all branches of the military and no current pre-clinical models of GWI take into consideration the incidence of mTBI, nor do current preclinical models of TBI consider the possible effects of subsequent toxic environmental exposures, we investigated these in our well-characterized mouse models of GWI and r-mTBI [4, 46, 73]. Our findings suggest that the chronic histological consequences of r-mTBI can be exacerbated by subsequent toxic chemical/environmental exposures in a multiple-hit hypothesis model.

## Methods

### Animals

Male and Female, C57BL/6 J mice (aged 10–12 weeks, 20–26 g, Jackson Laboratories, Bar Harbor ME) were housed under standard laboratory conditions (23 ± 1 °C, 50 ± 5% humidity, and 12-h light/dark cycle) with free access to food and water throughout the study. All procedures were carried out under Institutional Animal Care and Use Committee approval and in accordance with the National Institute of Health Guide for the Care and Use of Laboratory Animals.

### In vivo studies and schedule

**Fig. 1:**
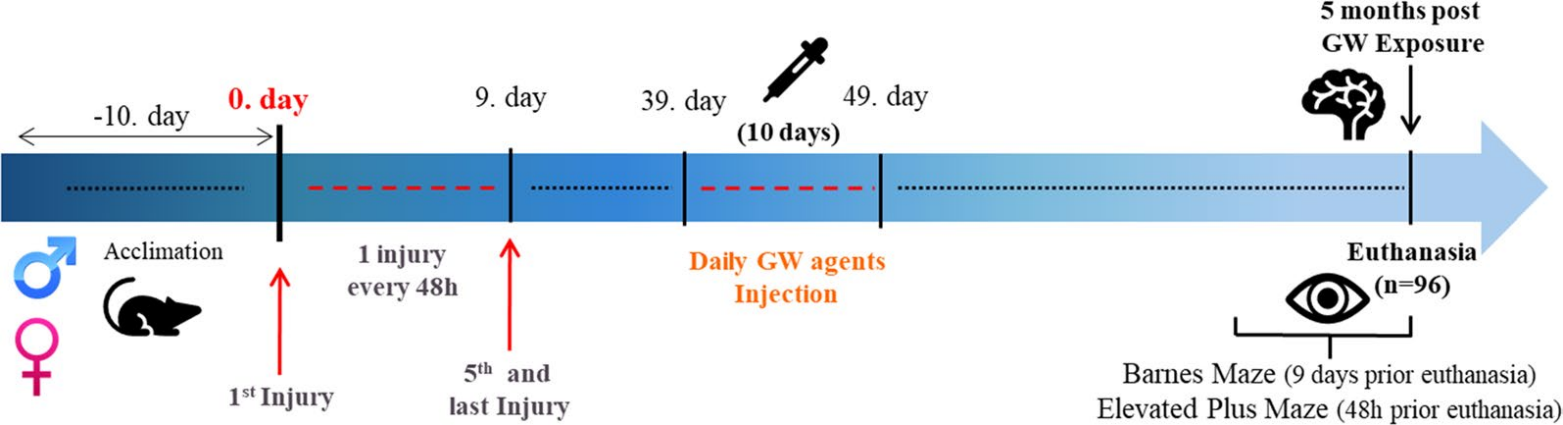
Outline of experimental schedule

A total of 96 mice were randomly assigned to four groups: 12 mice per group × 4 study groups (Sham/Vehicle or Sham/V; Sham/Gulf War toxic exposure or Sham/GW, r-mTBI/Vehicle or r-mTBI/V, r-mTBI/Gulf War toxic exposure or r-mTBI/GW, × 2 Sex (Male, Female). Studies were conducted using both male and female mice to determine the sex differences in response to r-mTBI and GW toxicant exposures. While this report focuses on the outcomes at 5 months post GW toxicant exposures, in a parallel study, mice are ageing to a 16 months’ timepoint following the same exposures, and so the weight and Elevated Plus Maze behavior data from those mice at the 5-month time point are included in this study. All 96 mice assigned to the 5 months’ time point were euthanized 24 h after completion of their last behavior test and the tissue was collected for biochemical and pathological analyses (Fig. 1).

### Mouse model of repetitive mTBI

The r-mTBI/sham procedure was administered to mice as previously described [45], with one mild injury or sham injury administered 5 times over a 9-day period. As described in our original publication on this model [45], the inter-injury interval was chosen to accommodate repeated injuries occurring within an asymptomatic window of vulnerability from the previous injury, as had been previously described in a rat model [39]. Mice aged 3 to 4 months were anesthetized with 1.5 L/min of oxygen and 3% isoflurane prior to r-mTBI or sham injury. The heads were shaved, and all animals were placed on a heating pad to maintain their body temperature at 37˚C. A 5 mm blunt metal impactor tip was retracted and positioned midway relative to the sagittal suture before each impact. The injury was triggered using the myNeuroLab controller at a strike velocity of 5 m/s, strike depth of 1.0 mm, and dwell time of 200 ms. At the end of the procedure, each animal was removed from the stereotaxic table and allowed to recover on a heating pad and, upon becoming ambulatory, was returned to its home cage. Sham injured animals underwent the same procedures and were exposed to anesthesia for the same length of time and frequency as the r-mTBI animals, but did not receive an injury, in order to control for the effects of the repeated anesthesia.

### Mouse model of GWI

One month following the last injury or anesthesia, the same animals were GW toxicant exposures or vehicle daily (controls), for 10 days, as described in our previous studies of GWI. [4, 73] Male and female mice (4/5 months of age) received GW toxicant exposures co-administered 0.7 mg/kg of PB (Thermo Fisher Scientific, Waltham, MA), and 200 mg/kg of permethrin (PER) (98.3% mixture of 27.2% cis and 71.1% trans isomers) (Sigma Aldrich, St. Louis, MO) in a single 50 μl intraperitoneal injection in dimethyl sulfoxide (DMSO) (Sigma Aldrich, St. Louis, MO) or DMSO alone (as control) daily for 10 consecutive days as previously reported [71, 73]. While the average daily PB and permethrin usage was provided as a guideline, the average exposure varied greatly by branch of service and by platoon (i.e., whether they were in forward unit and whether they were ordered to take PB by their commander or not and for Permethrin the uniforms were soaked every 5 days, but some did not do as instructed). Hence, we used data different parameters to estimate the relevance of the selected doses of each agent and choose to use the higher veteran’s exposure scenario. For PB, veterans were instructed to self-administer 90 mg per day (1.2 mg/kg) by taking one 30 mg tablet every 8 h [30]. Our proposed concentration in mice is 0.7 mg/kg (i.p.). The area under the curve (AUC) of oral PB administration in rats is 39 ng/mL/h (dose: 5.82 mg/kg) and in humans 231 ng/mL/h (dose: 0.85 mg/kg). To account for the difference in metabolism between mice and human the number is factorized (× 12), which results in a suggested i.p. dose of 0.9 mg/mL [48]. Less toxicological data is available for permethrin. However, there is a well conducted PBPK study in flight attendants which includes estimates of dermatological, oral and inhalational routes of entry [64]. Rat and human plasma data allowed the comparison of permethrin administration with mice. In a study from Amaraneni et al. steady state levels of cis and trans-permethrin were assessed in rats [8]. The applied dose in Sprague–Dawley rats was oral permethrin at 150 mg/kg and 16 mg/kg intravenous for 72 h to achieve steady-state levels at which point the steady state was reached at 55 ng/mL [8]. This was then compared to a PBPK study, where flight attendants have been exposed to permethrin via inhalation and potentially oral and dermal exposure. The estimated AUC in these flight attendants showed to be 16 ng/mL, which upon factorization due to the differing metabolism results in a comparable dose between human and rats [48]. These doses were chosen to mimic a high-level exposure that is similar to dose to mice showing adverse behavioral or pathological outcomes [4, 71, 73]. Overall, this shows that both selected doses are relevant exposure levels in humans.

### Assessment of behavioral outcomes

Behavioral outcomes were evaluated at 5 months post GW toxicant exposures using the Barnes maze (BM) in the same manner we described previously [45]. Researchers conducting the experiments were blinded to grouping, and the Ethovision XT system (Noldus, Wageningen, NL) was used to track and record the movement of each animal. Mice were given 90 s to locate and enter the target box, and they were required to remain in the target box for 30 s prior to retrieval. If the mice did not find the target box, they were gently guided to the target hole by the animal technician and remained there for 30 s prior to retrieval. For a period of 6 days, 4 trials were given per day, with mice starting from one of 4 cardinal points on each trial. On the 7th day a single probe trial lasting 60 s was performed with the mouse starting from the center of the maze and the target box removed. Spatial memory retention was measured by the distance traveled and time required for the animal to reach the target hole that used to contain the box.

We used a long short-term memory (LSTM) architecture to classify the search strategies used during Barnes maze acquisition and probe trials. Briefly described, the LSTM recurrent neural network was trained using 1024 Barnes maze trials from our previous work as part of the Chronic Effects of Neurotrauma Consortium (CENC). These trials were then classified continuously by a single trained, blinded observer who watched each recording and manually categorized the motion exhibited by the mouse as (i) spatial, (ii) serial, (iii) random, or (iv) no motion, throughout each trial using manual scoring in Ethovision XT. To help the LSTM learn to recognize the relevant features of the data sequences faster, several factors were pre-computed for every frame of data from the sequence of raw coordinates exported by Ethovision. This included the heading between the center point of mouse and the target hole, the distance between the center point of the mouse and the target hole, and the velocity of the mouse. Each trial was then processed into a continuous set of 120-frame sequences of data corresponding to 4 s of the trial. For training and validation, the complete dataset was artificially balanced by random selection of an equal number of sequences corresponding to spatial, serial, random or no motion. This model achieved 90% accuracy against a validation dataset of 42 trials that were kept separate from the training dataset. This model was then utilized to quantify the search strategy utilization in this study, in a blinded and unbiased fashion.

### Assessment of anxiety

Anxiety-like behavior was assessed 48 h prior to euthanasia using an elevated-plus maze which relies on the animal preference for dark enclosed arms over bright open arms. This task assesses the willingness of the mouse to explore the open arms of the maze which are fully exposed and at an elevated height. Time spent in the open arm is decreased in mice that exhibit anxiety-like behaviors. Researchers conducting the experiments were blinded to mouse group assignments and the Ethovision XT system (Noldus, Wageningen, NL) was used to track and record the movement of each animal.

### Histology

At 5 months post-GW toxicant exposures all 96 animals were anesthetized with isoflurane and perfused transcardially with phosphate-buffered saline (PBS), pH7.4 followed by PBS containing 4% paraformaldehyde. After perfusion, the brains were post fixed in a solution of 4% paraformaldehyde at 4 °C for 48 h. The intact brains were then blocked and processed as previously described [45]. For each group, sets of sagittal (lateral 0.2–0.4 mm) sections were cut. Sections were stained with Haematoxylin and Eosin and for Luxol fast blue/cresyl violet (LFB/CV) using standard histological protocols. Sets of adjacent sections were stained for glial fibrillary acid protein (GFAP, 1:20,000; Dako, Glostrup, Denmark, ZO334), ionized calcium binding adaptor molecule 1 (Iba1, 1:5000; Abcam, Cambridge, MA, ab5076), amyloid precursor protein (APP, 1:20,000; Millipore, Billerica, MA, MAB348), Oligodendrocytes 2 (Oligo2, 1:5000; Abcam, Cambridge, MA, ab109186), leucocyte common antigen 45 (CD45, 1:500, Cell Signaling, Danvers, M, #70,257). Tau immunohistochemistry was performed using the CP13 [pS202] monoclonal antibody, generously provided by Dr. Peter Davies, at a 1:1000 dilution. As a negative control for each antibody, a single section was processed for immunostaining without the inclusion of the primary antibody. Tissue sections were subjected to antigen retrieval with either heated trisethylenediaminetetraacetic acid (EDTA) buffer (pH-8.0) or citrate buffer (pH-6.0) under pressure for 7 min. Endogenous peroxidase activity was quenched with a 15 min H_2_O_2_ treatment (3% in water). Each section was rinsed and incubated with the appropriate blocking buffer (ABC Elite kit, MOM kit, Vector Laboratories, CA) for 20 min, before applying the appropriate primary antibody overnight at 4ºC. The diluted biotinylated secondary antibody from the ABC Elite Kit was then applied. Antibodies were detected using the avidin-peroxidase complex, after incubation with the chromogen 3,3-diaminobenzidine (DAB) peroxidase solution (0.05% DAB—0.015% H_2_O_2_ in 0.01 M PBS, pH 7.2) for 6–7 min and counterstained with hematoxylin. Immunofluorescence was performed with an antibody for p-tau CP13 (1:500). Prior to immunostaining, samples were deparaffinized in xylene and rehydrated through a gradient of ethanol solutions of decreasing concentrations (2 × 100%, 95%, 70%). Antigen retrieval consisted of heating slides in a citrate 9 solution (pH-6.0) under pressure, washed with PBS and transferred into a Sudan black solution (EMD Millipore, MA) (15 min) to inhibit autofluorescence. Before primary antibody treatment slides were blocked for 1 h with 10% donkey serum. The primary antibody for CP13, Oligo2, GFAP (Aves Labs, Inc, Davis, CA, GFAP) were applied on the slides and left overnight at 4ºC. The next day, donkey anti-Mouse IgG secondary antibody Alexa Fluor 488, was applied for CP13. Slides were mounted with ProLong Gold Antifade DAPI Mount. Fluorescent imaging was performed using a confocal microscope (LSM 800 Zeiss) at 20 × and 63 × magnification. Z-stacks were recorded for every image and orthogonal projections were obtained to enable a 3D representation of the picture. Non-fluorescent samples were visualized with a bright field microscope (BX60, Leica, Germany) and digital images were visualized and acquired using a MagnaFire SP camera (Olympus, Tokyo, Japan).

### Immunohistochemical quantification

For each animal (n = 8/12 per group) sagittal sections were stained and analyzed by an observer blinded to experimental conditions using ImageJ software (US National Institutes of Health, Bethesda, MD, USA). Images were separated into individual color channels (hematoxylin counter stain and DAB) using the color deconvolution algorithm [56]. Three non-overlapping areas of 100 μm^2^ for the body of the corpus callosum (CC) were randomly selected within which the area of GFAP/Iba1/CD45 immunoreactivity was calculated and expressed as a percentage of the field of view (lateral 0.2–0.4 mm). Four non-overlapping areas of 150 μm^2^ between layer III and IV in the primary somatosensory cortex, and 3 non-overlapping areas of 100 μm^2^ in the hippocampus were randomly selected within which the area of GFAP/Iba1/CD45 immunoreactivity was calculated and expressed as a percentage of the field of view (lateral 0.2–0.4 mm). The numbers of APP-positive profiles were counted and totaled in three non-overlapping areas of 200 μm^2^ while the number of Oligo2^+^ was averaged from three non-overlapping areas of 200 μm^2^ within the body of the CC. Using ImageJ software, the average thickness of the CC was calculated as previously described [46].

### Statistical analysis

All behavioral and pathological data were analyzed using GraphPad Prism 9.0 (San Diego, USA). Data were tested for normality using the Shapiro–Wilk W Test; when not normally distributed, the data were transformed using square root or natural log transformation. If the data were still not normal after transformation, non-parametric methods were used for analysis. Behavioral experiments were analyzed using 2-way ANOVA, repeated measure ANOVA and PERMANOVA for search strategy quantification. Repeated-measures ANOVA was used to compare performance during the 6 days of acquisition of the Barnes maze between the matching injury groups when the data were normally distributed. Potential sphericity violations were corrected by adjusting degrees of freedom for all repeated-measures effects by using the Greenhouse–Geisser estimate for epsilon. Probe, elevated plus maze and quantitative histologic parameters were analyzed with Two-way ANOVA, with a Tukey’s *post-hoc* correction for multiple comparisons, unless indicated. Only P values < 0.05 were considered to be statistically significant and are indicated by an asterisk in the figures.

## Results

### Barnes maze: acquisition

**Fig. 2:**
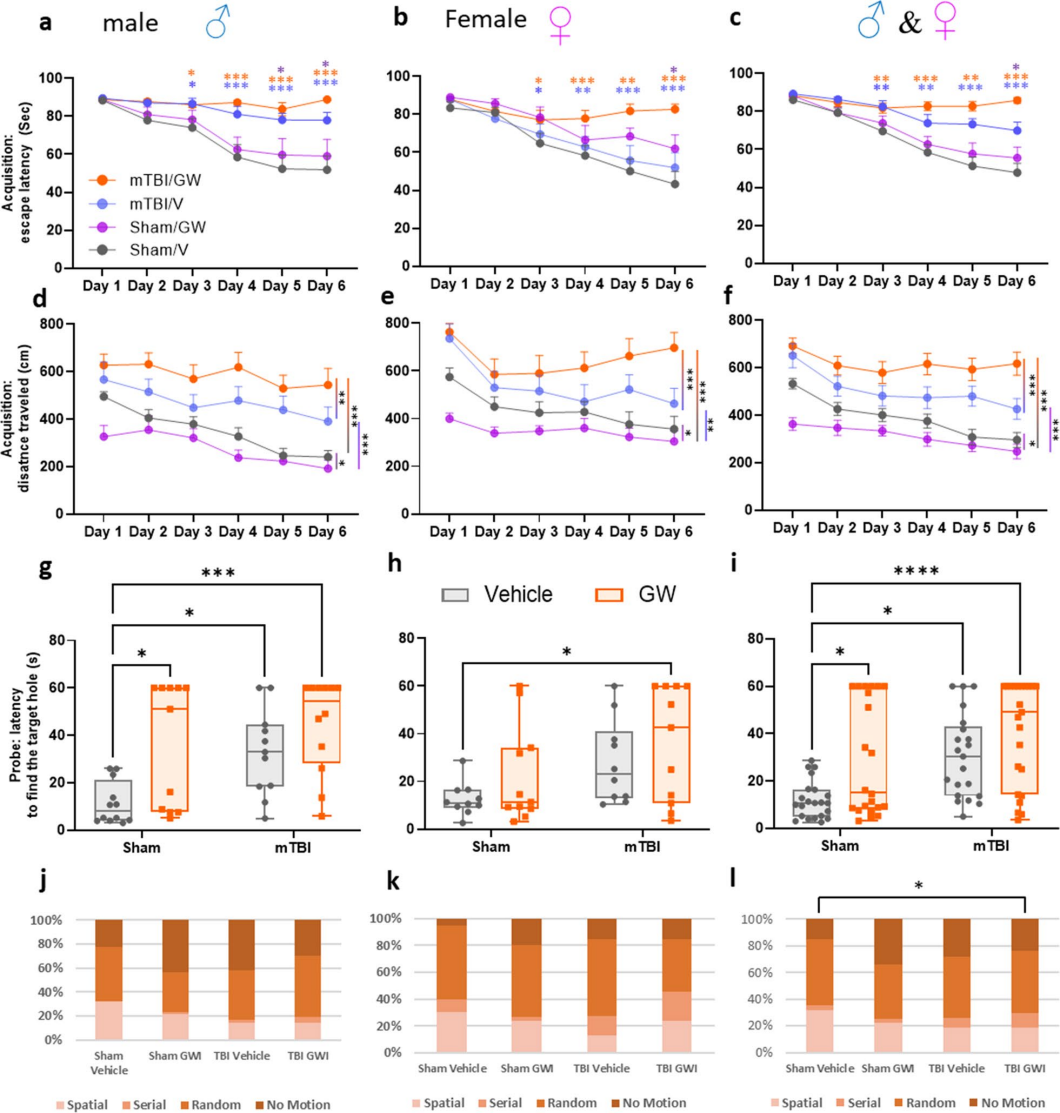
Assessment of neurobehavioral change at 5 months Post‐GW toxic exposure and for both Sexes. **a** Learning deficits were evaluated during the 6 days of acquisition. Since no sex interactions were observed for the outcome reported from the Barnes maze test, samples were consolidated across sexes for all following experiments (**c**, **f**, **i**). During the acquisition testing, both injured groups spent more time on the table before escaping into the target hole compared with their respective controls (**c**). The r-mTBI/GW group spent the longest time on the platform (**c**, Wilcoxon signed rank test, *P* < 0.0001) and traveled the longest distance (**f**, *P* < 0.0001; repeated‐measures ANOVA). The r-mTBI/GW group was also found to perform worse than the r-mTBI/V group on day 4,5 and 6 for escaping the maze and travelled greater distance on the maze for the 6 days of acquisition (**c**, Wilcoxon signed rank test, *P* < 0.01; **f**, *P* < 0.0001; repeated‐measures ANOVA). The performance of the Sham/GW was better than both r-mTBI groups for the time to escape the maze and but worse than the Sham/V on day 4, 5 and 6 (**c**, Wilcoxon signed rank test, *P* < 0.01). The Sham/GW traveled the shortest distance of all groups (**f**, *P* < 0.01; repeated‐measures ANOVA). Evaluation of spatial memory retention (probe) using the Barnes maze at 5 months post GW exposure pooling Male and Female mice (**g–l**). Both r-mTBI groups had a greater latency to reach the target zone than the Sham/V or Sham/GW (**i**, *P* < 0.006; Two-way ANOVA, Tukey's multiple comparisons test). The Sham/GW group also had a longer latency to reach the target hole; Sham/GW vs. Sham/V *P* < 0.0377 (**i**). No difference was observed between the Sham/GW and the r-mTBI/V or between the r-mTBI/V and r-mTBI/GW. Data are presented as mean ± SEM, Two‐way analysis of variance with Tukey's post hoc test; *P* > 0.05; each symbol represents 1 mouse *N* = 10/12 per group per Sex, solid symbol: male; empty symbol: female). Quantification of search strategy utilization in the probe trial of the Barnes Maze (**j–l**). There was a narrowly significant overall effect of GW treatment and mTBI on the raw counts of search strategy utilization in the probe trial (*P* = 0.05; PERMANOVA, 199 permutations). Pair-wise comparisons with Holm-Bonferroni corrections for multiple comparisons showed that when the sexes were combined, r-mTBI/GW mice (**l**) had a different search strategy utilization pattern than Sham/V mice (*P *< 0.04; PERMANOVA, 199 permutations)

No sex effect was observed for the outcome reported from the Barnes maze test (Fig. 2a, b, d, e, g, h). Sexes were subsequently combined for the remaining analyses (Fig. 2c, f, i). The dataset for latency to escape was not normally distributed and thus did not satisfy the assumptions required for a repeated-measure ANOVA. The Wilcoxon signed rank test was used to test for the daily correlation between each group. At 5 months post GW toxicant exposures, the r-mTBI/GW group performed the worst and showed no improvement in escape latency performance (88.3 ± 2.2 s on day 1 to 85.9 ± 2.4 s on day 6). In contrast, the remaining three groups all showed improvement over the 6 days of training with the r-mTBI/V improving the least (89.2 ± 2.1 s on day 1 to 69.9 ± 1.4 s on day 6), followed by the Sham/GW (88.3 ± 2.4 s on day 1 to 55.3 ± 5.8 s on day 6) and the Sham/V (85.9 ± 1.4 s on day 1 to 47.6.9 ± 4.9 s on day 6) performing the best. On day 6 of the acquisition trial, the r-mTBI/V performed better in the maze than the mTBI/GW mice (r-mTBI/V vs. r-mTBI/GW; 69.9 ± 1.4 s vs. 85.90 ± 4.7 s; P < 0.017; Wilcoxon signed rank test). Both of the injured groups travelled on average a longer distance to reach the target box when compared to their respective sham groups (Fig. 2d–f), (r-mTBI/V vs. Sham/V, P < 0.0001; r-mTBI /GW vs. Sham/V, *P* < 0.0001; repeated-measures ANOVA). This deficit in performance of the r-mTBI/GW group was also apparent when compared to the r-mTBI/V group (r-mTBI/GW vs. r-mTBI/V, *P* < 0.0001; repeated-measures ANOVA). The Sham/GW group travelled continually less distance during the entire acquisition period (Sham/V vs. Sham/GW, *P* < 0.05; repeated-measures ANOVA) but spent more time on the table than the Sham/V.

### Barnes maze: probe

The probe trial analysis of the average time to reach the target hole, revealed that the r-mTBI/GW mice performed the worst, requiring on average 40.53 ± 4.7 s to reach the target hole, followed by the r-mTBI/V (30.51 ± 3.8 s), the Sham/GW (29.13 ± 5.1 s), and the Sham/V (11.89 ± 1.6 s), (Fig. 2i). The Sham/GW group had a longer latency to reach the target hole when compared to the Sham/V (Sham/GW vs. Sham/V *P* < 0.0377, Two-way ANOVA, Tukey's multiple comparisons test). No difference was observed between the Sham/GW and the r-mTBI/V or the r-mTBI/GW. However, both mTBI groups performed worse than the Sham/V group (r-mTBI/V or r-mTBI/GW vs. Sham/V *P* < 0.006, Two-way ANOVA, Tukey's multiple comparisons test).

Primary search strategy analysis was performed using an LSTM neural network to quantify the search strategy utilization of each mouse, placing each frame of the trial into one of four categories (spatial, serial, random and no motion) and counting those frames until the mouse finds the target hole (primary strategy quantification). Using the raw quantification of each search strategy category to construct a distance matrix of 4 dimensional coordinates for each mouse, a PERMANOVA analysis of all groups found that the search strategy differences were narrowly significant overall (*p* = 0.05). The greatest differences were between r-mTBI/GW and Sham/V mice (Fig. 2j–l). r-mTBI/GW mice utilized a spatial search strategy for only 18% of the probe trial until finding the target hole, versus 31% for Sham/V mice. Conversely, r-mTBI/GW mice exhibited a greater reliance on systematic serial searching to find the target hole than Sham/V mice (11% vs 4% respectively). While Sham/V and r-mTBI/GW mice exhibited similar amounts of random motion prior to finding the target hole (49% and 46% respectively), Sham/V mice spent less time motionless prior to finding the target hole than r-mTBI/GW mice (15% and 29% respectively). Pairwise comparisons showed the r-mTBI/GW to Sham/V difference in strategy utilization to be significant after correcting for multiple comparisons. (Fig. 2l, p = 0.03; PERMANOVA with Holm-Bonferroni corrections for multiple comparisons, 199 permutations).

### Elevated plus maze

**Fig. 3:**
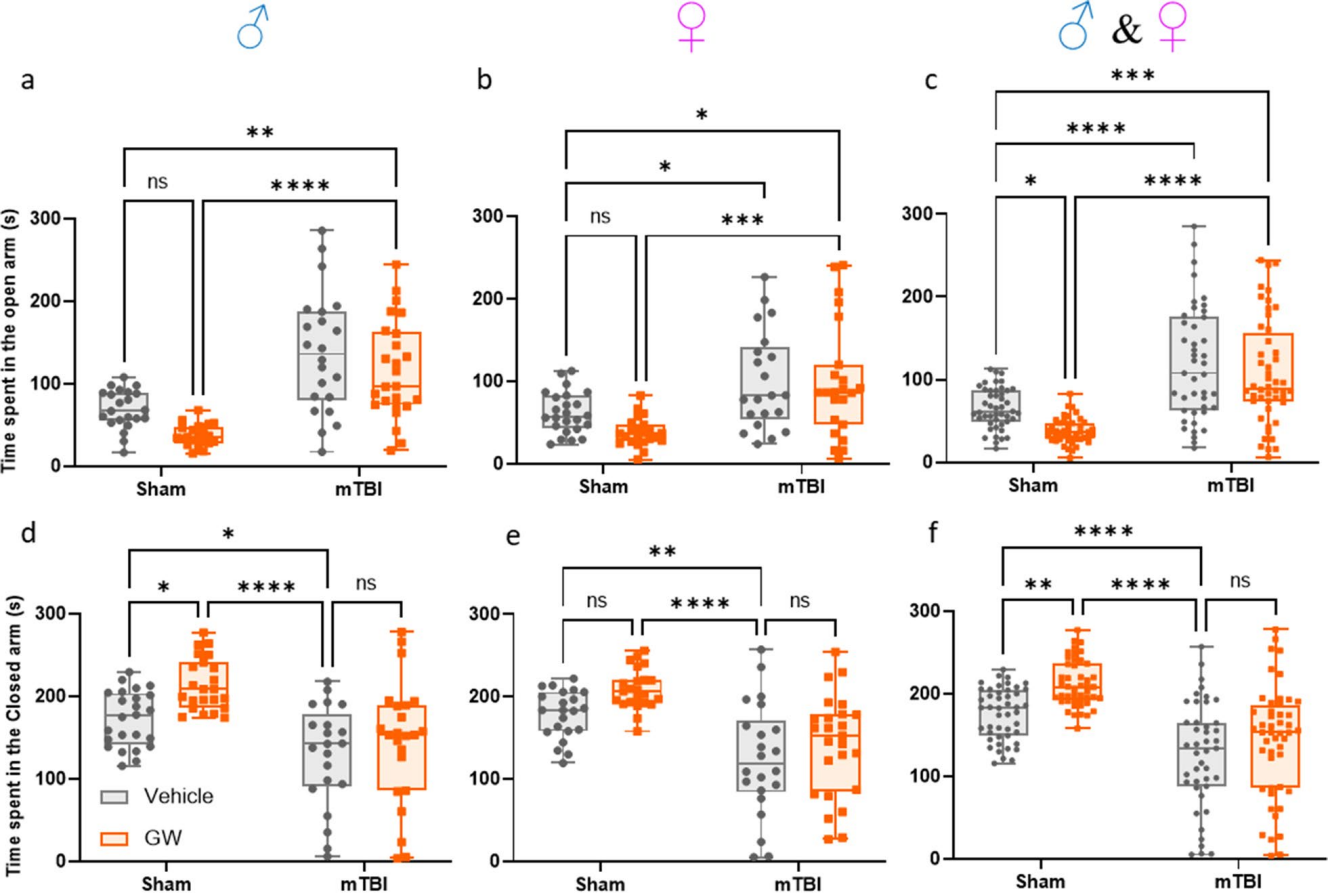
Assessment of risk like behavior at 5 months Post‐GW toxic exposure and for both Sexes. **a**, **b** For both Sex, mice in both mTBI groups developed an increased risk-taking behavior by spending more time in the open arms of the elevated plus maze when compared with their respective sham control, (**c**, *** P < 0.0001 and *P* < 0.00001 Two-Way ANOVA-Tukey). Sham/GW exposed animals performed the opposite of the mTBI groups displaying a greater anxiety by spending less time in the open 'arms and more time in the closed arms (**c**, **f**, *P* < 0.05 Two-Way ANOVA-Tukey). A three-Way ANOVA analysis for the time spent in the open arm revealed a Sex, Injury and Exposure effect: *F*_(1, 175)_ = 4.47 P=0.0358; *F*_(1, 175)_ = 74.62 *P *< 0.0001; *F*_(1, 175)_ = 7.59 *P* = 0.0065 respectively, but no interaction effect. A three-Way ANOVA analysis for the time spent in the closed arm revealed an Injury and Exposure effect: *F*_(1, 179)_ = 63.19 *P *< 0.0001; *F*_(1, 179)_ = 11.36 *P* = 0.0009; but no Sex effect *F*_(1, 179)_ = 0.1525 *P* = 0.696. Data are presented as Whiskers plots: Min to Max. Each symbol represents 1 mouse *N *= 22/24 per group per Sex)

Following probe testing at 5 months post GW toxic exposure, the anxiety and risk-taking behaviors of each animal were tested with the elevated-plus maze (Fig. 3). Since no sex interactions were observed for any of the behavioral tests, sexes were combined for analyses (Fig. 3a, b, d, e). Both sham groups spent a smaller amount of time in the open arm than the mTBI groups (Sham/V, 61.95 ± 5.1 s; Sham/GW, 46.7 ± 4.0 s; r-mTBI/V, 100.5 ± 3.3 s; r-mTBI/GW 99 ± 9.7 s; *P* < 0.0005; Two-way ANOVA, Tukey's multiple comparisons test). The analysis of the time spent in the closed arm revealed that both sham groups spent more time in the closed arm than the mTBI group, with the Sham/GWI group staying the longest time in the closed arm (Sham/V, 174.2 ± 7.1 s; Sham/GW, 203.4 ± 7.2 s; r-mTBI/V, 138.5 ± 12.3 s; mTBI/GW 145 ± 9.7 s Two-way ANOVA, Tukey's multiple comparisons test). The Sham/GWI group resulted in significantly reduced time spent in the open arms of the maze, consistent with increased anxiety, while both injured cohorts spent more time in the open arm which is consistent with lack of anxiety. For both outcomes measured, amount of time spent in closed and open arms, the Sham/GW showed an increased anxiety when compared to the Sham/V (*P* < 0.01 Two-Way ANOVA-Tukey: Fig. 3c, f). No difference was observed between the r-mTBI/V and r-mTBI/GW groups (*P* > 0.05).

### Physiological appearance at euthanasia:

Since we have previously reported lipidomic and metabolic dysfunction in our pre-clinical models of GWI [5, 72], consistent with lipid changes detected in blood samples from GWI veterans [23], we recorded the body weight for each animal at 5-months post GW toxicant exposures **(**Additional file 1: Fig. S1**)**. The animals of both r-mTBI groups had lower body weights when compared to the sham control groups. A sex effect was present with the male mice weighing more than the female mice. Next, we examined the effect of GW toxicant exposures in altering leukocyte dynamics in peripheral lymphoid tissues. It is also known that exposure to r-mTBI can lead to chronic immune dysfunction [11, 35, 50, 73]. As such, the weights of the spleen and thymus were examined at euthanasia (Additional file 1: Fig. S2). An injury effect was present whereby the animals receiving r-mTBI had a larger spleen weight. A sex effect was also present for the weights of the spleen and thymus due to the lower body weight of female mice.

### Effect of GWI and/or r-mTBI on astrogliosis in the cortex and hippocampus:

**Fig. 4:**
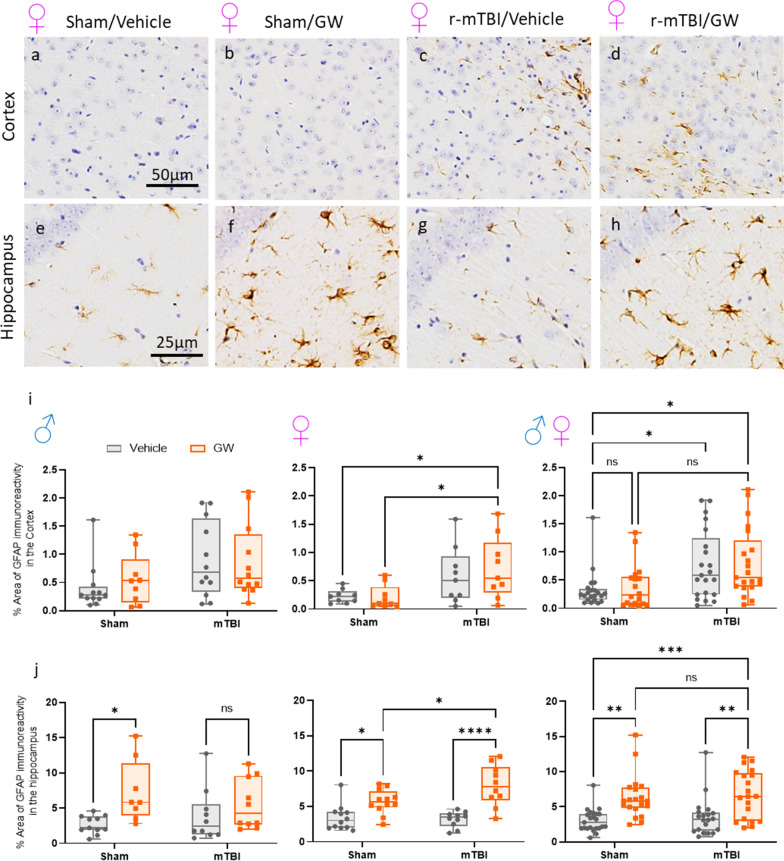
Evaluation of GW toxic exposure on astroglial activation (glial fibrillary acidic protein [GFAP]) at 5 months after mTBI in the superficial layers of the cortex and in the Hippocampus. In both brain regions, healthy astrocytes with quiescent morphology were observed in sham vehicle animals (**a**, **e**). Gulf War exposure had no effect overall on the cortex, but an injury effect was observed in the cortex of female animal (**a–d**, I, * P < 0.05, Two-Way ANOVA-followed by Tukey *post hoc *comparisons). A three-Way ANOVA analysis revealed a Sex and Injury effect: Sex *F*_(1, 73)_ = 4.742 *P *< 0.05; Injury *F*_(1, 73)_ = 14.22 *P *< 0.0003; Exposure *F*_(1, 73)_ = 0.2545, *P* > 0.05. In the hippocampus (CA1/CA3) both male and female sham/GW exposed animals showed a moderate astrogliosis with variable degree of thickened cell processes and hypertrophic cell soma. (**e–h**, **j** *P < 0.005; Two-Way ANOVA-followed by Tukey *post hoc *comparisons) but no injury effect was reported. Three-Way ANOVA summary revealed only an exposure effect for the dataset: Sex *F*_(1, 75)_ = 0.1208 p=7291; *F*_(1, 75)_ = 0.4167, *P* = 0.5206; Treatment *F*_(1, 75)_ = 31.50 *P *< 0.0001. Data are presented as Whiskers: Min to Max. Show all points; symbol represents 1 mouse *N *= 8/12 per group per Sex)

An increased astrogliosis was noted at 5 months post GW toxicant exposures in our previous studies [73], therefore, the same brain regions were evaluated to determine whether prior r-mTBI could interact with a subsequent exposure of GW toxicant exposures. In the superficial layers of the cortex, there was no evidence of increased GFAP immunoreactivity in mice subjected to GW toxicant exposures. The expected injury effect was observed between either injured group (Fig. 4a–d, r-mTBI/V, 0.74 ± 0.15; r-mTBI/GW, 0.79 ± 0.06) compared to the Sham/V (0.31 ± 0.08; *P* = 0.028 and *P* = 0.015 respectively), but not when compared to Sham/GW (P value). Consistent with our previous report [46, 71], immunostaining for GFAP revealed evidence of a mild reactive astrogliosis in the hippocampus of the animals with GW toxicant exposures but no r-mTBI effect was observed [Fig. 4. Sham/GW vs. Sham/V, 6.49 ± 0.81, 3.02 ± 0.37 (*P* < 0.001) and r-mTBI/GW, 6.75 ± 0.20 vs. r-mTBI/V, 3.53 ± 0.35 (*P* < 0.002)]. A three-way ANOVA revealed a Sex effect in the cortex with the female mice showing less astrogliosis than their male counterparts (*P* = 0.03), although no Sex effect was overserved in the hippocampus. For both brain regions, no Injury x Exposure interaction was observed.

### Effect of GWI and/or r-mTBI on microgliosis in the cortex and hippocampus

**Fig. 5:**
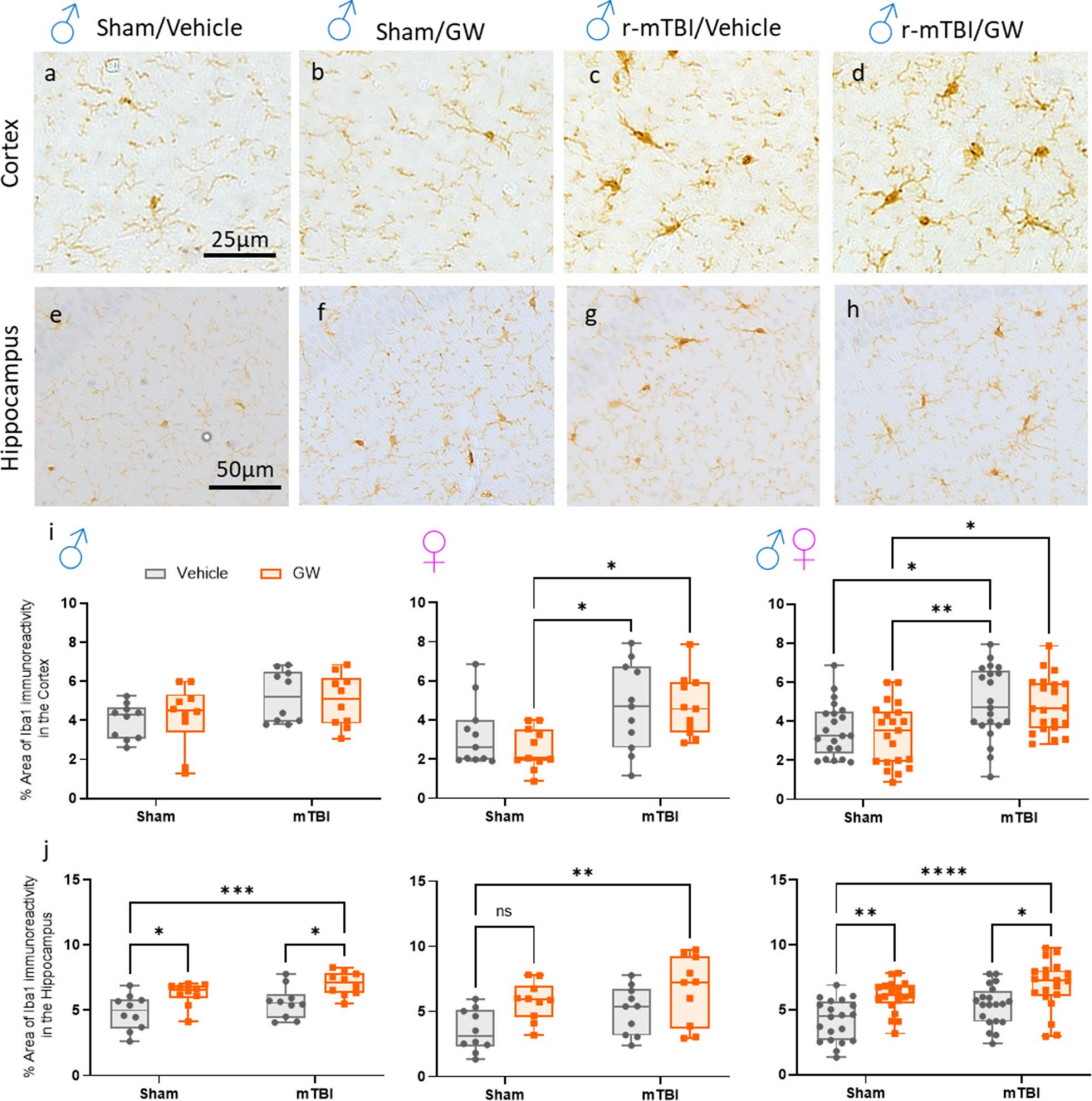
Evaluation of GW toxic exposure on microglial activation (Ionized calcium binding adaptor molecule 1[Iba-1]) at 5 months after r-mTBI in the superficial layers of the cortex and Hippocampus. Healthy microglia with quiescent morphology were observed in sham vehicle animals. At 5-month post injury, only an injury effect was observed in the cortex when both Sex were combined between the Sham/GW and both r-mTBI groups (**c, d**, I, *P* < 0.05, Two-Way ANOVA-followed by Tukey *post hoc *comparisons). A three-Way ANOVA analysis revealed a Sex and Injury effect: *F*_(1, 76)_ = 6.066 P=0.0160; *F*_(1, 76)_ = 17.87, *P *< 0.0001; *F*_(1, 76)_ = 0.2471 P=0.6206. In the hippocampus (CA1/CA3) only males from the Sham/V and r-mTBI/GW animals and female from the r-mTBI/GW showed a moderate microgliosis with variable degree of thickened cell processes and hypertrophic cell soma. **e–h, j**. When both sexes were combined, this effect was exacerbated with GW toxic exposure (J) (*P* < 0.05, Two-Way ANOVA-followed by Tukey *post hoc *comparisons). Three-Way ANOVA summary revealed an Injury and Exposure effect for the dataset: Sex *F*_(1, 72)_ = 3.499, *P* = 0.0655; Injury *F*_(1, 72)_ = 7.944 P=0.0062; Exposure *F*_(1, 72)_ = 22.53 *P *< 0.0001. Data are presented as Whiskers: Min to Max. Show all points; symbol represents 1 mouse *N *= 10 per group per Sex)

The same brain regions were analyzed with the marker Iba1, a pan-microglial marker whose expression increases with microglial activation. The morphological characteristics of (Iba1 +) cells were relatively similar in the cortex of the Sham/V animals with the appearance of a quiescent microglia cell (Fig. 5a, b). In the cortices of both injured groups a slight but noticeable increase in size of their cell body was seen compared to controls. Overall, no GW effect was observed in the cortex of all groups. For mice subjected to TBI, immunostaining for anti-Iba-1 revealed clusters of activated microglia with thick cell bodies in the region of the cortex underlying the impact (Fig. 5a–d, i, r-mTBI/V, 4.94 ± 0.27 vs Sham/V 3.61 ± 0.35 (*P* = 0.034) r-mTBI/GW 4.85 ± 0.16 vs Sham/GW 3.37 ± 0.84 (*P* = 0.012)). As with astrogliosis, GW toxicant exposures effect was only observed in the hippocampus, with both exposure groups showing a mild microgliosis at 5 months post GW toxic exposures (Fig. 5e–h, j, Sham/GW, 6.02 ± 0.22 vs Sham/V 4.19 ± 0.66 (*P* < 0.002) r-mTBI/GW 6.86 ± 0.18 vs r-mTBI/V 5.34 ± 0.24 (*P* = 0.017)). Once more, a Sex effect was observed only in the cortex (*P* = 0.016) with the female mice expressing less microgliosis than their male counterparts. For both brain regions, no Injury x Exposure interaction was observed. In addition, we also examined the same brain regions with cluster of differentiation receptors 45 (CD45) to assess microgliosis and potential infiltration of CD45-positive leukocytes (Additional file 1: Fig. S3). However, no exposure or injury effect was observed across each group.

### GW toxicant exposures increase gliosis in the corpus callosum, a brain region known to be chronically inflamed post r-mTBI

The corpus callosum is a brain region susceptible to insult from r-mTBI [33, 67] and GW [62] toxicant exposure. In our animal model, r-mTBI results in ongoing white matter degeneration in the corpus callosum (CC) which we have documented across the mouse lifespan [47]. Given this, we analyzed the integrity of the CC and the degree of inflammation present in our cohorts. To determine the thickness of the corpus callosum, a Luxol fast blue stain was performed which stains the white matter of the brain. In comparison to the thickness of the CC in the Sham/V, the thickness was decreased by 15% in the Sham/GW, 35% in the r-mTBI/V, and by 40% in the r-mTBI/GW. An injury effect was present with the injured groups showing a greater decrease in thickness of the CC compared to non-injured counterparts.

To investigate the extent of axonal injury, tissue was stained for amyloid precursor protein (APP). Positive staining for APP was present in the r-mTBI/V and r-mTBI/GW groups but no positive staining was found among sham animals, indicating an injury effect. An effect of GW toxicant exposures was also noted as the density of APP -immunoreactive profiles per unit area in the body of the CC of the r-mTBI/GW was greater than in the r-mTBI/V group.

To gain insight into whether GW toxic exposures could exacerbate the degree of inflammation in the corpus callosum among animals with and without a history of r-mTBI, we investigated three different markers of glial cells, GFAP a marker of astrocytes, Iba-1 a marker of microglia, and CD45 a marker of activated microglia. We also evaluated the presence of oligodendrocytes through Olig2. An increase in oligodendrocytes was noted with the GWI/Vehicle displaying 35.7 cells per 200 μm^2^, the r-mTBI/V displaying 41.7 cells per 200 μm^2^, and the r-mTBI/GW displaying 58.1 cells per 200 μm^2^. Overall, there was an effect of injury and GW toxicant exposure, but not sex.

Reactive astrogliosis and microgliosis was observed only in the r-mTBI/V group and r-mTBI/GW group. No difference in astrogliosis and microgliosis was noted between the Sham/V and Sham/GW. A GW toxic exposure and an injury effect, but no sex effect, was present among staining for GFAP and Iba-1. The morphological characteristics of the astroglia and microglial cells were relatively similar across each group, with a primed morphology characteristic of a mid-aged mouse brain. However, a greater degree of microglial activation was observed among animals with GW toxicant exposures. The Sham/GW displayed greater staining for CD45 than the Sham/V. Likewise, the r-mTBI/GW was more reactive to the CD45 antibody than the r-mTBI/V. Overall, there was an effect of injury and exposure but not sex.

### Phospho Tau deposits were found in oligodendrocytes after r-mTBI and GW toxicant exposures:

**Fig. 6:**
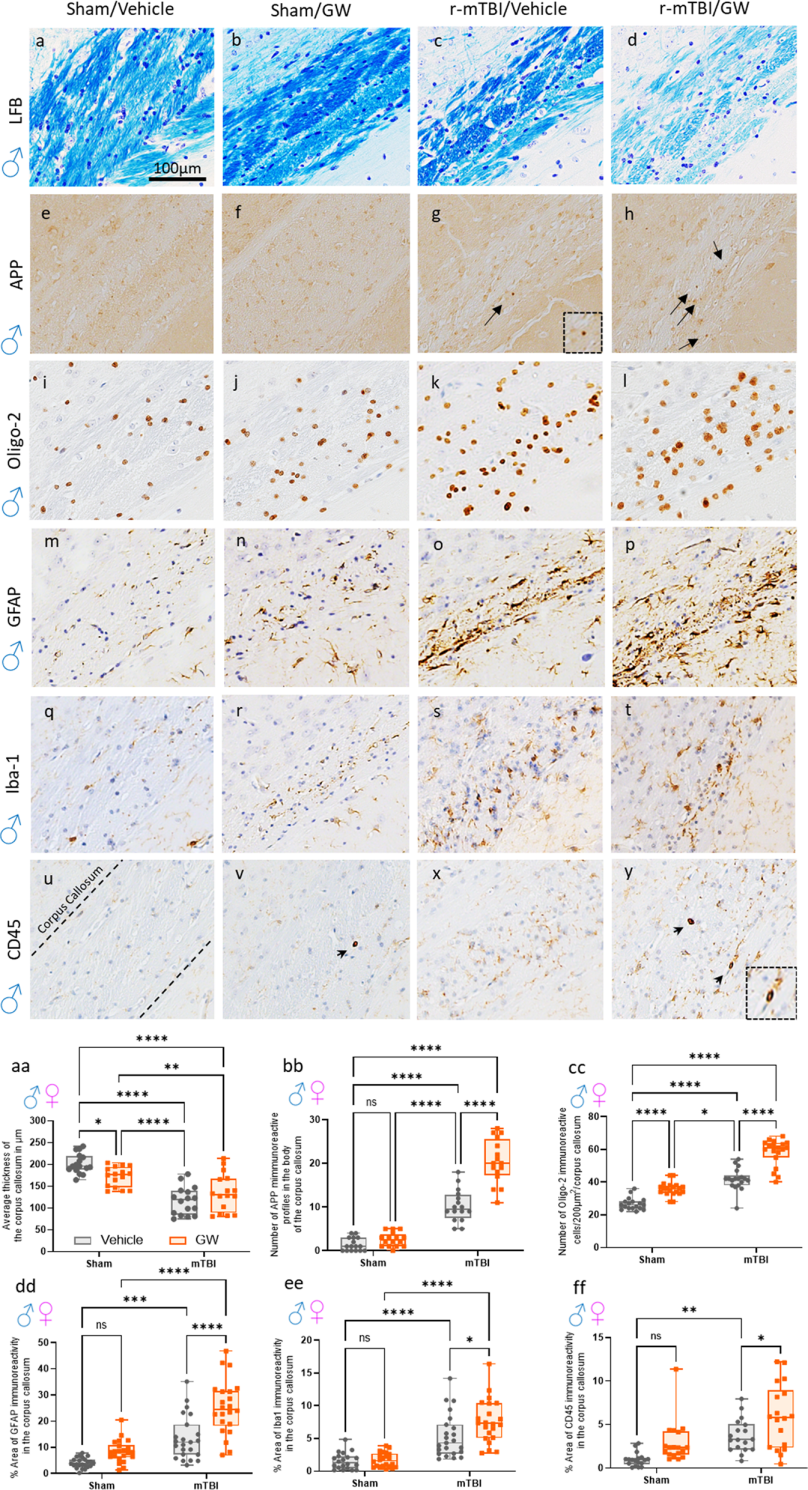
Evaluation of the extent of white matter integrity at 6 months after injury in the corpus callosum. **a**–**d** Luxol fast blue/cresyl violet (LFB/CV) staining indicated changes in white matter integrity with a thinning of the corpus callosum (CC) by approximately 15% in the GW treated group, 40% in the r-mTBI group and 35% in the r-TBI/GW group (*P* < 0.0001) when to compared to Sham/V. **aa** Quantitative analysis of the thickness of the corpus callosum. (*P < 0.05, Two-Way ANOVA-followed by Tukey post hoc comparisons). A three-Way ANOVA analysis revealed a Sex and Injury effect: *F*_(1, 56)_ = 13.58 P=0.0005; *F*_(1, 56)_ = 87.58, *P *< 0.0001; but not Treatment effect *F*_(1, 56)_ =  0.68, *P* = 0.41. **e**, **h** Granular axonal profiles stained positively with the axonal injury marker (amyloid precursor protein [APP]) were predominantly seen in both r-mTBI groups within the CC when compared to their respective sham control. **bb** The density of APP-immunoreactive profiles per unit area in the body of the CC of the r-mTBI/GW group was greater than in the r-mTBI/V group (r-mTBI/V, 10.25 ± 1.0 vs r-mTBI/GW, 20.44 ± 2.3 axonal profiles/body of CC, *P* < 0.0001; Data are presented as mean ± standard error of the mean). Magnification of the boxed inset represents the area indicated by the arrow. A three-Way ANOVA analysis revealed no Sex effect *F*_(1, 56)_ = 2.6, *P* = 0.1, an Injury effect: *F*_(1, 56)_ = 290.3 *P *< 0.0001 and a Treatment effect; *F*_(1, 56)_ = 52.16 *P *< 0.0001. **i**, **l** The number of Oligodendrocytes cells was counted using the Oligo2 pan marker, a transcription factor that is expressed in both mature oligodendrocytes as well as oligodendrocyte precursor cells. **bb** At 6 months post injury an increase of the number of Oligo2^+^ cells were observed in the GW group with 35.7 cells per 200 µm^2^, followed by the r-mTBI group with 41.7 cells per 200 µm^2^ while the r-mTBI/GW group showed the highest number of positive cells 58.1 cells per 200 µm^2^ (*P *< 0.0001, Two-Way ANOVA-followed by Tukey post hoc comparisons). A three-Way ANOVA analysis revealed no Sex effect *F*_(1, 67)_ = 0.13, *P* = 0.7, an Injury effect: *F*_(1, 67)_ = 208.0 *P *< 0.0001 and a Treatment effect; *F*_(1, 56)_ = 90.88, *P *< 0.0001. **m–p** Strong reactive astrogliosis was observed in the body of the CC in both r-mTBI groups when compared to the r-sham group while no difference was observed between the sham/V and the sham/GW. **dd** Quantitative analysis of glial fibrillary acidic protein (GFAP) staining in the body of the CC (Sham/V, 4.9 ± 0.3% vs Sham/GW, 8.4 ± 2.5%; *P* > 0.05; r-mTBI/V 13.3 ± 5.0% vs r-mTBI/GW, 24.6 ± 7.0%; *P *< 0.0001). A three-Way ANOVA analysis revealed a Sex effect *F*_(1, 67)_ = 0.13 *P *< 0.0001, an Injury effect: *F*_(1, 67)_ = 208.0 *P *< 0.0001 and a Treatment effect; *F*_(1, 56)_ = 90.88 *P *< 0.0001. **q–t** Iba-1 immunostaining showed moderate immunoreactivity in both TBI while no difference was observed between the Sham/V and the Sham/GW. **ee** Quantitative analysis of Iba-1 staining in the body of the CC (Sham/V, 1.54 ± 1.0% vs Sham/GWI, 1.79 ± 0.8%; *P* > 0.05; TBI/V 5.3 ± 1.5% vs r-mTBI/GW, 7.6 ± 1.4%; P=0.019). A three-Way ANOVA analysis revealed a Sex effect *F*_(1, 79)_ = 24.66 *P *< 0.0001, an Injury effect: *F*_(1, 79)_ = 98.5 *P *< 0.0001 and a Treatment effect; *F*_(1, 79)_ = 7.06 P=0.0095. **u–y** A second immunostaining with cluster of differentiation receptors 45 (CD45) probed for activated microglia and peripherical macrophage. **ff** Quantitative analysis of CD45 staining in the body of the CC (Sham/V, 0.97 ± 0.2 vs Sham/GW, 2.9 ± 0.49%; *P* > 0.05; r-mTBI/V 3.7 ± 0.07% vs r-mTBI/GW, 5.9 ± 0.12%; *P* = 0.042). A three-Way ANOVA analysis revealed no Sex effect *F*_(1, 64)_ = 0.32, *P* > 0.05, an Injury effect: *F*_(1, 64)_ = 23.97 *P *< 0.0001 and a Treatment effect; *F*_(1, 79)_ = 12.44, *P* = 0.008. Tissue sections were counterstained with hematoxylin. Data are presented as Whiskers: Min to Max. Show all points; symbol represents 1 mouse *N *= 8/12 per group per Sex)

**Fig. 7:**
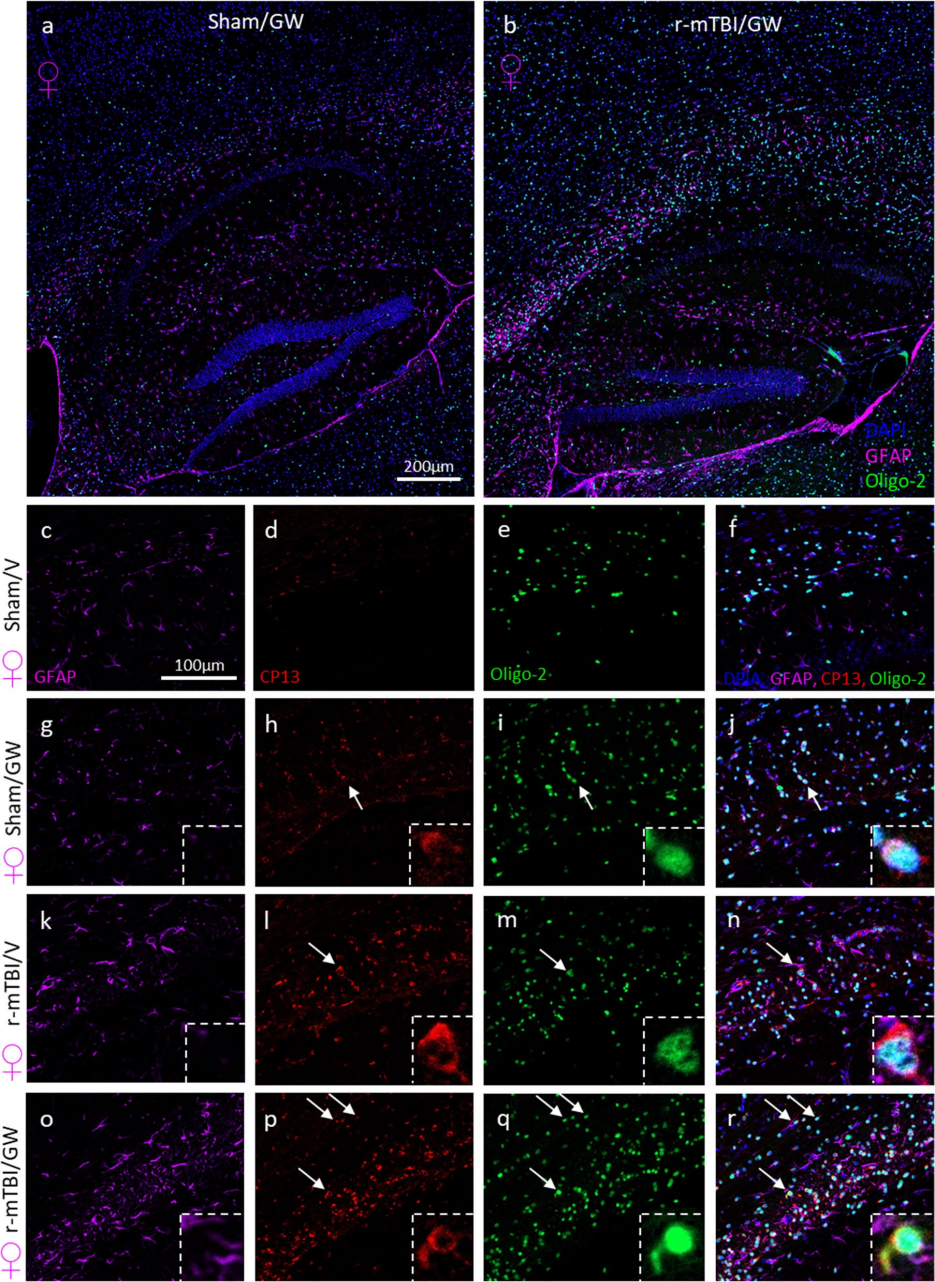
Evidence of p-Tau pathology in oligodendrocytes in female mice exposed to GW toxic exposure with and without a history of r-mTBI. Representative images of immunofluorescence staining of the astrocyte marker GFAP, a marker of oligodendrocyte Oligo-2, and phosphorylated Tau CP13 (pSer-202) in the cortex/CC/hippocampal of female mice euthanized at 5 months post GW agent treatment (**a**, **b**). No difference between male and female mice was observed. Mice with a history of r-mTBI prior GW agent exposure qualitatively show an increased reactive astrogliosis in the three sub regions of the corpus callosum and in the hippocampus. In the body of the corpus callosum, co-labeling for p-Tau CP13 and Oligo-2 was found in all r-mTBI and GW groups (**g**–**r**). No Tau immunofluorescence was observed in the Sham/Vehicle animals (**d**). p-Tau staining in the r-mTBI/GW group was always markedly increased and more abundant than any of the other groups (**p**). In the magnified insets (**j**, **h**, **r**) the nuclear marker of oligodendrocytes Oligo2 establishes the co-localization with the aggregation of cytoplasmic p-tau CP13 characteristic of an “olicap” structure

This set of experiments was used to evaluate the extent of the white matter damage at chronic time points post-GW toxicant exposure with a focus on the corpus callosum, a brain region we have previously shown to be affected by both GW toxicant exposures and repetitive mTBI. Triple-labeling immunofluorescence was carried out using a chicken polyclonal antibody to astrocytes (GFAP), a rabbit antibody to Oligodendrocytes (Oligo2) and mouse antibody to p-Tau (Ser-202); CP13. For both Sexes, as previously reported in Fig. 5, TBI increased the density of oligo2 positive cells in both the corpus callosum and the cortex (Fig. 6). An increased reactive gliosis was also observed consistent with our immunohistochemical analysis in the corpus callosum of the injured mice and in the hippocampus of the GW toxicant exposures groups (Fig. 7). No p-Tau deposits were seen in the Sham/V animals (Fig. 7). Phospho-tau immunoreactive profiles were distributed throughout the corpus callosum, with a distinctive and characteristic perinuclear location in oligo-2 + cells, forming caps or comma-like deposits: “olicap”. The distribution of p-tau deposit was present in the corpus callosum of the Sham/GW, r-mTBI/V and r-mTBI/GW groups, although deposits were more evident in both injured groups (Fig. 7p).

### Gulf War toxicant exposure exacerbates TBI-dependent p-tau deposits in the corpus callosum without affecting the hippocampus:

Exposure to r-mTBI has been associated with the development of phosphorylated tau (p-tau). To explore the effect of r-mTBI and GW toxicant exposures on tau pathology, CP13, a marker of p-tau, was examined in the body of the CC at 5 months post GW toxicant exposures. GW toxicant exposures alone did not have an effect on the accumulation of p-tau, however, a GW toxicant exposures effect was observed among the r-mTBI/GWI animals. The presence of CP13 was also evaluated in the CA3 region of the hippocampus, a region known to be involved in tau hyperphosphorylation. Each group displayed a physiological somatodendritic accumulation of CP13 in the hippocampus. GW toxicant exposures did not affect the accumulation of p-tau (Additional file 1: Fig. S4).

## Discussion

To our knowledge, there have been no pre-clinical studies published evaluating the impact of secondary insults such as a “chemical stressor” on animals with a history of r-mTBI. Multiple toxicant and chemical stressors may contribute to the etiology of GWI, including potential sources such as burn pits in forward operating bases, nerve agents from destroyed munition sites, organophosphates insecticides and PB used prophylactically against potential nerve agent exposure [26, 40, 65]. Indeed, burn pits remain a potential source of toxicant exposure for Veterans involved in more recent operations including Operation Enduring Freedom (OEF) and Operation Iraqi Freedom (OIF) and among Military personnel serving after September 11, 2001 [60]. Additional research is needed to understand the long-term health outcomes for Veterans with r-mTBI and how environmental exposures, such as burn pits and other toxic chemicals, are associated with chronic disease mortality [31]. For the purposes of our study, we utilized a model of GWI consisting of a combined exposure to PB and PER and our results indicate that GW toxicant exposures post r-mTBI leads to worse neuropathological outcomes than in comparison to GW toxicant exposures or repetitive mTBI alone, specifically in the corpus callosum. These findings were reflected in our behavioral studies which showed that mice with both r-mTBI and GW toxicant exposures showed the greatest impairment in learning and memory.

Using the Barnes maze to assess spatial learning and memory, we found that GW toxicant exposures worsened the outcome for r-mTBI animals, with r-mTBI/GW mice exhibiting higher escape latencies on the Barnes maze during the final three days of acquisition testing than r-mTBI/V mice. This also coincided with a significant increase in the distance the r-mTBI/GW mice traveled during the final day of acquisition testing. The increased latency to escape from the Barnes maze could in part be due to increased anxiety-like behavior in GW exposed mice observed during elevated plus maze testing, but this GW-dependent decrease in the time spent in the open arms was only observed in uninjured (sham) GW mice. Previous studies have shown that this model of GW exposure can produce anxiety-like behavior in mice at chronic, but not acute, post-exposure time points [4]. In this study, r-mTBI resulted in disinhibition and a greater amount of time spent in the open arms relative to sham animals, regardless of GW toxicant exposures. This finding is consistent with our previously published studies [44, 46, 47]. TBI-dependent disinhibition appeared to nullify any GW toxicant exposures-dependent increases in anxiety, arguing against anxiety as a primary driver of r-mTBI/GW increases on latency observed in our Barnes maze testing.

The probe trial revealed that Sham/GW, r-mTBI/V and r-mTBI/GW mice all had a higher latency to locate the target hole than Sham/V mice. Interestingly, stratification by sex showed that the differences between Sham/V and Sham/GW as well as Sham/V and r-mTBI/V were only significant in male mice, suggesting a possible protective effect in female mice. The prevalence and nature of GWI within the female population of Gulf War veterans warrants further study, and the hormonal status may affect the clinical presentation as well [19]. In a recent study female veterans of the Gulf War have self-reported poorer health than their male counterparts [29], but whether this extends to GWI and reflects a true disparity with our pre-clinical model remains to be determined [21]. Previous studies have also shown a delayed effect in this model with GW exposure resulting in spatial memory impairments in the Barnes maze at 5 months post exposure, without affecting short-term spatial memory or learning [73]. In order to better understand the nature of the deficits observed in the Barnes maze, we also used an artificial neural network to quantify the search strategies used during the primary portion of the probe trial until the mouse found the target hole. This analysis revealed that Sham/V mice exhibited the greatest proportion of spatial strategy utilization than any other group, and there was a significant shift in strategy utilization within the r-mTBI/GW mice. Mice within the r-mTBI/GW group spent less time utilizing spatial strategies, more time utilizing systematic search strategies, and more time stationary on the maze prior to finding the target hole. This indicates that the combined effect of r-mTBI and GW resulted in greater reliance on non-spatial strategies, correlating with an increase in the latency to find the target hole independent of changes in anxiety or disinhibition observed in the elevated plus maze.

Astrogliosis is known to be present in human cases of GWI as demonstrated by a study which found increased autoantibodies to GFAP in the blood of veterans with GWI [6]. Furthermore, an imaging study evaluating the brains of veterans with GWI noted abnormal lactate uptake, an indicator of active astroglia [53]. When investigating astrogliosis in our model of GWI and r-mTBI, we found a mild increase in GFAP signal among r-mTBI mice in regions of the cortex underlying the impact site. However, on this occasion, we did not detect any cortical astrogliosis in the Sham/GW mice indicating that the PB/PER exposure alone may not have influenced astrogliosis in this cohort. When compared to their male counterparts, female mice showed less GFAP signal in the cortex. This may be attributed to the fact that cycling of hormone levels throughout the estrous cycle is known to influence GFAP immunoreactivity [75]. Regarding the astrogliosis in the hippocampus, our past studies have shown an increase in GFAP among GW treated animals [34, 71]. However, no effect of r-mTBI was noted. The lack of r-mTBI effect may be attributed to the fact that GFAP expression is greatest in astrocytes that are proximal to the injury site, while the hippocampus is deeper in the brain tissue.

Microgliosis is known to occur following insult to the CNS and is common to the pathology of mTBI. Past investigations of microgliosis in mouse models of GWI have not found evidence of microgliosis occurring in the cortex but have shown microgliosis to be present in the hippocampus [35, 50, 51]. Our findings reflected this as microgliosis in the cortex was only noted among animals who received r-mTBI, not among those solely with GW toxicant exposures. In the hippocampus however, our study found microgliosis to be present among animals with GW toxicant exposures. Among animals who received r-mTBI, GW toxicant exposure exacerbated microgliosis. Considering the inflammation present in the hippocampus demonstrated by astrogliosis and microgliosis, it could be hypothesized that this mild inflammation contributes to the cognitive dysfunction noted in GWI. In fact, studies have linked mild inflammation in the hippocampus as one of the factors contributing to cognitive dysfunction in GWI [12, 52].

White matter abnormalities have been linked to several of the symptoms associated with GWI in veterans, such as widespread pain, fatigue, and cognitive dysfunction [54]. Specifically, brain imaging studies of veterans with GWI have indicated lower white matter integrity and the presence of axonal pathology in white matter tracks involving pain processing, fatigue, and reward processing [54]. Other indications of poor white matter health have been noted in the brains of veterans with GWI. Imaging studies have found degrees of demyelination, inflammation, edema, and changes in axonal morphology. Furthermore, studies have shown an interaction between peripheral inflammation and white matter inflammation in GWI, reflective of the systemic nature of the illness [61, 62].

Studies evaluating the white matter in animal models of GWI have been limited to investigating oligodendrocyte involvement in a rat model of GWI. In this model, rats were co-administered corticosterone and di-isopropyl fluorophosphate (DFP), a sarin gas analog and acetylcholinesterase inhibitor. The study found that exposure to acetylcholinesterase inhibitors disrupts neuron-glia interactions and concluded that oligodendrocyte pathology plays a significant contribution to GWI pathophysiology [9]. Specifically, it was noted that corticosterone exposure and co-exposure of corticosterone and DFP was associated with a higher frequency of oligodendrocytes in the corpus callosum when compared to control. In our mouse model of GWI and r-mTBI, we also observed an increase in oligodendrocytes in the CC of animals who were exposed to r-mTBI and GW toxicant exposures. An increase in oligodendrocytes was noted between Sham/GWI animals and Sham/V animals. Interestingly, the group displaying the greatest increase in Olig2 + cells was the r-mTBI/GWI group, indicating a synergistic effect of GW toxic exposures and r-mTBI on Olig2 + cell count. When investigating the co-localization of p-tau and oligodendrocytes, we found the presence of p-tau among animals to be most evident in the r-mTBI/GW animals. In vitro approach using human induced pluripotent stem cells from the blood of veterans with GWI also revealed elevated levels of total and phosphorylated tau in neurons exposed to low-level organophosphate pesticides and nerve agents suggesting a potential mechanistic explanation for the memory loss suffered by veterans with GWI [68]. Further studies are warranted to understand the effect of such pathology in the CC and whether this ongoing neuroinflammation is the cause or effect of this increased number of Olig2 + cells.

In addition to studying oligodendrocyte involvement, we evaluated the effect of GW toxicant exposure and r-mTBI on the integrity and pathology of the CC through analyzing the thickness and extent of axonal injury. The CC is particularly sensitive to insult from mTBI in humans and our preclinical models, with thinning of the body of the CC occurring post injury [44, 47, 63]. This finding of decreased CC width post mTBI was reflected in our findings. Notably, GW toxicant exposures alone also caused a decrease in the thickness, and when in combination with TBI the thickness was further decreased. The finding that GW toxicant exposures affect CC integrity is reflected in human imaging studies that have demonstrated impaired interhemispheric communication and microstructural integrity GWI patients [16, 17, 62]. As previously mentioned, axonal pathology has been detected in imaging studies of Veterans with GWI [18]. Axonal pathology was also noted in our study, as r-mTBI mice showed an increase in APP, an indicator of axonal injury, in the CC. We also found that GW toxicant exposures may exacerbate this pathology, as more APP immunoreactivity was found in the CC of the r-mTBI/GW group when compared to the mTBI/V group.

Inflammation in the CC is known to occur post head injury, as demonstrated by the presence of reactive astrogliosis and microgliosis in animal models of mTBI [43, 44]. Our current study further supported these findings as both astrogliosis and microgliosis was noted in the CC of injured animals. Of note, the greatest degree of microgliosis was observed in animals with a history of r-mTBI and GWI. Reactivity to CD45, a marker of activated microglia, was also found to be highest among r-mTBI/GW treated animals, indicating that GW toxicant exposures may exacerbate r-mTBI pathology. Recovery after mTBI varies among the patient population with some patients displaying better outcome measures than others. These differences in outcomes may be attributed to pre-existing conditions, genetic factors or concurrent polytrauma [36, 74]. However, less attention has been paid to the effect of secondary insults during the chronic phase of mTBI on outcome measures, and the consequences are not well understood.

The insight and limitations of this animal study needs to be interpreted with care. One major problem is the very different responses seen in mice in the way they absorb and eliminate chemicals, in the way the chemicals are broken down or stored in the body and differences in body size, diet, lifespan, organ’s function that also cause mice to respond differently from humans. Another limitation of this study is that it focused on PB and PER to the exclusion of other military service-related toxic exposures in general, including prophylactic medications, pesticides, organophosphates, toxic industrial chemicals, airborne hazards and burn pits. In many instances, the extent of human exposure is difficult to quantify. In addition to the variety of exposures, it is not known how these various toxicants would have interacted with the association of high physiological stress to replicate GW theater condition. As a result, animal models that include PB, PER or DEET were also developed to include physical stress [3, 5, 58] or exposure to corticosterone [50] (a rodent stress hormone) as a potential contributor to GWI [20]. But even with this more complex models, current approaches have yielded mixed success in the translation of therapies to the clinical arena for GWI. Finally, some investigators have emphasized that organophosphate chemicals are a key component in the development of GWI leading to chronic neuroimmune disorder in rodents when combined with other exposures [20, 37, 38, 41, 42, 50]. While PB is not an organophosphate, it does share the property of being an acetylcholinesterase inhibitor. To reduce the number of variables that could confound the outcomes, we choose to restrict our study design by only adding two variables to our current model of GWI: r-mTBI and Sexes. Despite the limitations when translating from laboratory TBI models to human populations in these two models we describe components that are clinically relevant.

**Fig. 8:**
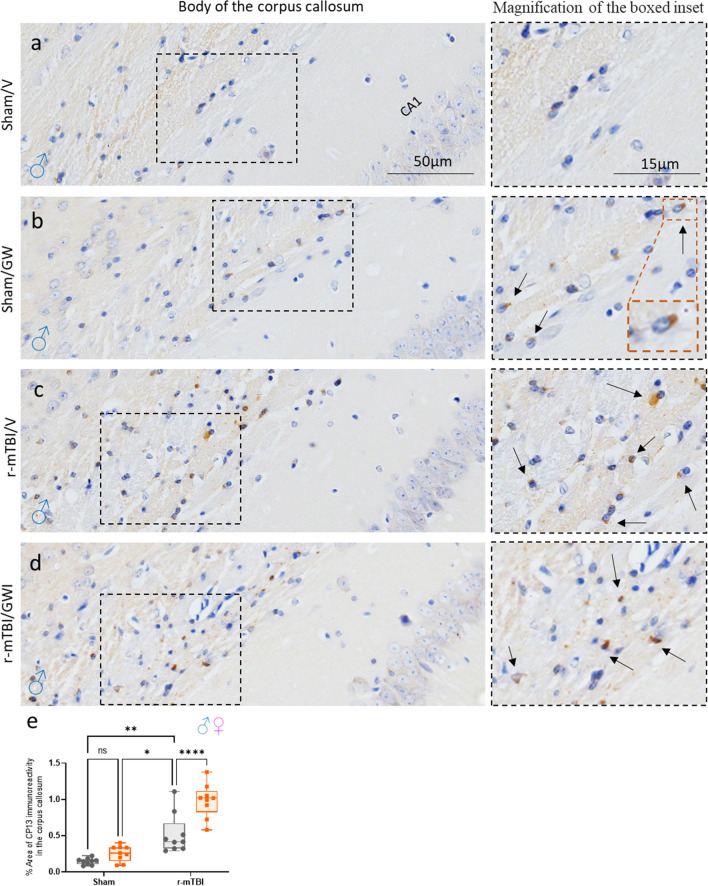
Immunohistochemical assessment of p-tau pSer-202 (CP13) in the body of the corpus callosum, at 5 months post GW toxic exposure. **a**–**d** GW treatment alone had no effect overall on the accumulation of p-Tau CP13 in the body of the corpus callosum (Sham/V, 0.14 ± 0.001 vs Sham/GWI, 0.25 ± 0.003; *P* > 0.05) but a GW treatment effect was observed in the animals previously exposed to r-mTBI (r-mTBI/V 0.53 ± 0.13% vs r-mTBI/GWI, 0.96 ± 0.15%; *P *< 0.0001, *P* < 0.05, Two-Way ANOVA-followed by Tukey post hoc comparisons). A three-Way ANOVA analysis revealed no Sex but a Treatment and an Injury effect: Sex *F*_(__1, 28)_ = 0.013 *P *< 0.90; Injury *F*_(__1, 28)_ = 98.58 *P *< 0.0001; Treatment *F*_(__1, 28)_ = 24.18 *P *< 0.0001. Data are presented as mean SEM; Data are presented as Whiskers: Min to Max. Show all points; symbol represents 1 mouse *N *= 4 per group per Sex)

To our knowledge, this is the first published preclinical study that has evaluated the effects of GW toxicant exposures in a model of r-mTBI. We found that the GW toxicant exposures enhanced the TBI-dependent pathological outcomes, indicating that GW toxicant exposures may exacerbate r-mTBI pathology. Specifically, ongoing white matter pathology in the CC was significantly impacted by the addition of GW toxicant exposures post r-mTBI. Worsened cognitive deficits and slower rate of learning was also noted among animals with both a history of r-mTBI and GW toxicant exposures suggesting a compromise in cognitive reserve after multiple insults**.** This implicates that clinically, veterans with GWI and a history of r-mTBI may demonstrate more symptoms indicative of perturbation in subcortical white matter brain region and impaired learning and memory function [16, 17]. More importantly, this study demonstrates that exposure to environmental factors post injury can modify the outcomes and pathobiology of traumatic brain injury (Fig. 8).

## Conclusion

**Fig. 9:**
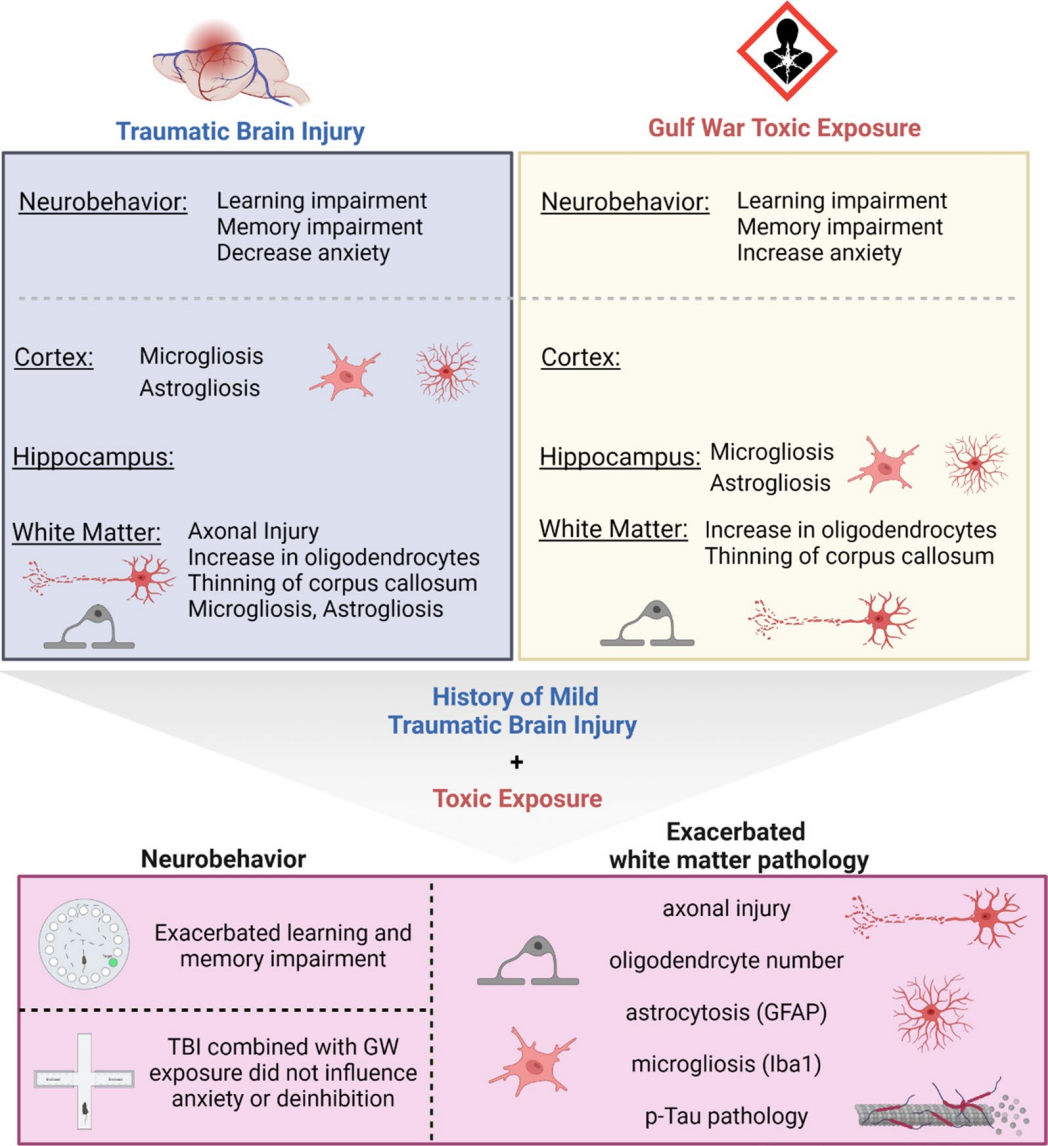
Summary of the study

In this mouse model of mild traumatic brain injury and Gulf War Illness we have established the first evidence that subsequent exposure of various toxic substances can influence the chronic behavioral and neuropathological consequences of mTBI. The findings from this study summarized in Fig. 9 also suggest that both exposure to mTBI and GW toxicants appears to lead to higher risk of chronic health effects in mice and likewise be an etiological factor influencing the trajectory of symptoms seen in GW veterans. It is crucial that TBI researchers and health organizations recognize how exposure to toxic substances can significantly alter mTBI neuropathology. There is a need for further studies to interrogate the association between r-mTBI, toxic exposures and subsequent illnesses in civilians and military veterans.

## Supplementary Information

**Additional file 1: Figure S1.** Body weight at euthanasia. (**a**)There was a sex effect (p<0.0001) with the male weighting more than their female counterpart. No difference in body weight was recorded between either the Sham/V vs Sham/GWI groups or between mTBI/Vehicle vs mTBI/GW groups. An injury effect was observed only in the males with both injured groups weighting less than the control groups; Sham/V 36.4g vs. mTBI/V 32.9g *P < 0.02 and Sham/V vs. mTBI/GWI *P < 0.01; data are presented as Interleaved box & whiskers plots - Min to Max, 3 Way analysis of variance with Tukey's post hoc test; each symbol represents 1 mouse; n=10/12 per group). (**b**) Results of the 3 Way ANOVA tables for the weight at euthanasia.

**Additional file 2: Figure S2.** Spleen and Thymus weight in percentage of body weight at euthanasia. (**a**) Representative images taken from male mice at 5 months post GW treatment are shown to illustrate the spleen and thymus size. (**b**, **c**) For both tissue, a Sex effect observed due to the lower body weight of female mice (P<0.0001, 3 Way-ANOVA). (**b**, **d**) An injury effect was also observed with the injured animals having in general a larger spleen weight compared to their body weight (P=0.0341; Three-Way-ANOVA). (**d**, **e**) Results of the 3-Way ANOVA tables for the weight at euthanasia. Data are presented as Interleaved box & whiskers plots - Min to Max, 3 Way analysis of variance.

**Additional file 3: Figure S3.** Immunostaining with cluster of differentiation receptors 45 (CD45) probing activated microglia and peripherical macrophage. at 5 months after mTBI in the hippocampus. (**a**–**d**) No changes in neurogenesis were observed at 5 months post injury or GW treatments by evaluating the % of area stained and morphology of the microglias. Qualitatively, there was a trend for an increased number of cells of with a doughnut-shaped blob (**d**, magnified inset), possibly as an infiltrating T cell as they were also observed within and along the blood vessels (**d**, arrows).

**Additional file 4: Figure S4.** Immunohistochemical assessment of p-tau pSer-202 (CP13) in CA3 region of the hippocampus and dentate gyrus at 6 months post injury. (**a**–**d**) Gulf War treatment alone had no effect overall on the accumulation of p-Tau CP13 in the CA3 sub region of the hippocampus and in the dentate gyrus. All experimental groups exhibited physiological somatodendritic accumulation of CP13 in the hippocampus. Each symbol represents 1 mouse. (k,l) 3-Way ANOVA summary tables. Data are presented as Whiskers plots: Min to Max. Show all points; symbol represents 1 mouse n=4 per group per Sex).

## Data Availability

The datasets used and/or analyzed during the current study available from the corresponding author on reasonable request.

## Abbreviations

AUC: Area under the curve
BM: Barnes maze
C57BL/6: A common inbred strain of laboratory mouse
CA1: Cornu Ammonis 1, the first region in the hippocampal circuit, from which a major output pathway goes to layer V of the entorhinal cortex
CA3: Cornu Ammonis 3, it receives input from the mossy fibers of the granule cells in the dentate gyrus
CC: Corpus callosum
CD45: Cluster differentiation 45
CENC: Chronic effects of neurotrauma consortium
CV: Cresyl violet
DFP: Di-isopropyl fluorophosphate
DVBIC: Defense and veterans brain injury center
DMSO: Dimethyl sulfoxide
EPM: Elevated plus maze
Fig: Figure
GFAP: Glial fibrillary acidic protein
GW: Gulf war
GWI: Gulf war illness
IBA1: Ionized calcium binding adapter molecule 1
LFB: Luxol fast blue
LSTM: Long short-term memory
Mg: Milli gram
ml: Milli liter
Olig2+: Oligodendrocyte marker
OEF: Operation enduring freedom
OIF: Operation Iraqi freedom
PER: Permethrin
r-mTBI: Repetitive mild traumatic brain injury
PB: Pyridostigmine bromide
TBI: Traumatic brain injury
