# Supplementary figures and images for: Impact of gulf war toxic exposures after mild traumatic brain injury
Source: Acta Neuropathol Commun. 2022 Oct 18;10:147. doi: 10.1186/s40478-022-01449-x (PMC9580120; doi:10.1186/s40478-022-01449-x)

## Slide 1
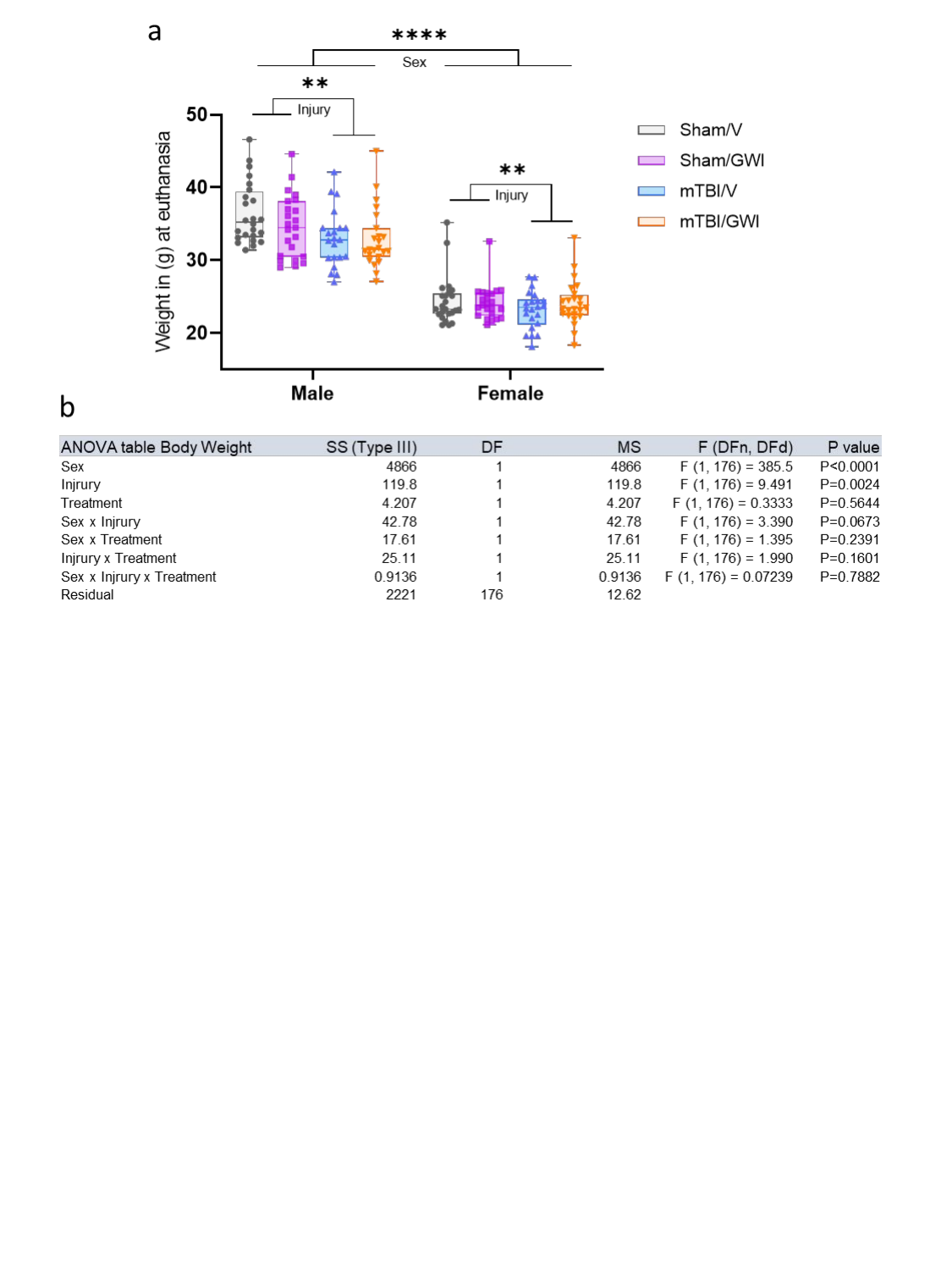

Supplement: Supplementary file 1 — Additional file 1: Figure S1. Body weight at euthanasia. (a)There was a sex effect (p<0.0001) with the male weighting more than their female counterpart. No difference in body weight was recorded between either the Sham/V vs Sham/GWI groups or between mTBI/Vehicle vs mTBI/GW groups. An injury effect was observed only in the males with both injured groups weighting less than the control groups; Sham/V 36.4g vs. mTBI/V 32.9g *P < 0.02 and Sham/V vs. mTBI/GWI *P < 0.01; data are presented as Interleaved box & whiskers plots - Min to Max, 3 Way analysis of variance with Tukey's post hoc test; each symbol represents 1 mouse; n=10/12 per group). (b) Results of the 3 Way ANOVA tables for the weight at euthanasia. [file 40478_2022_1449_MOESM1_ESM.pptx]

## Slide 1
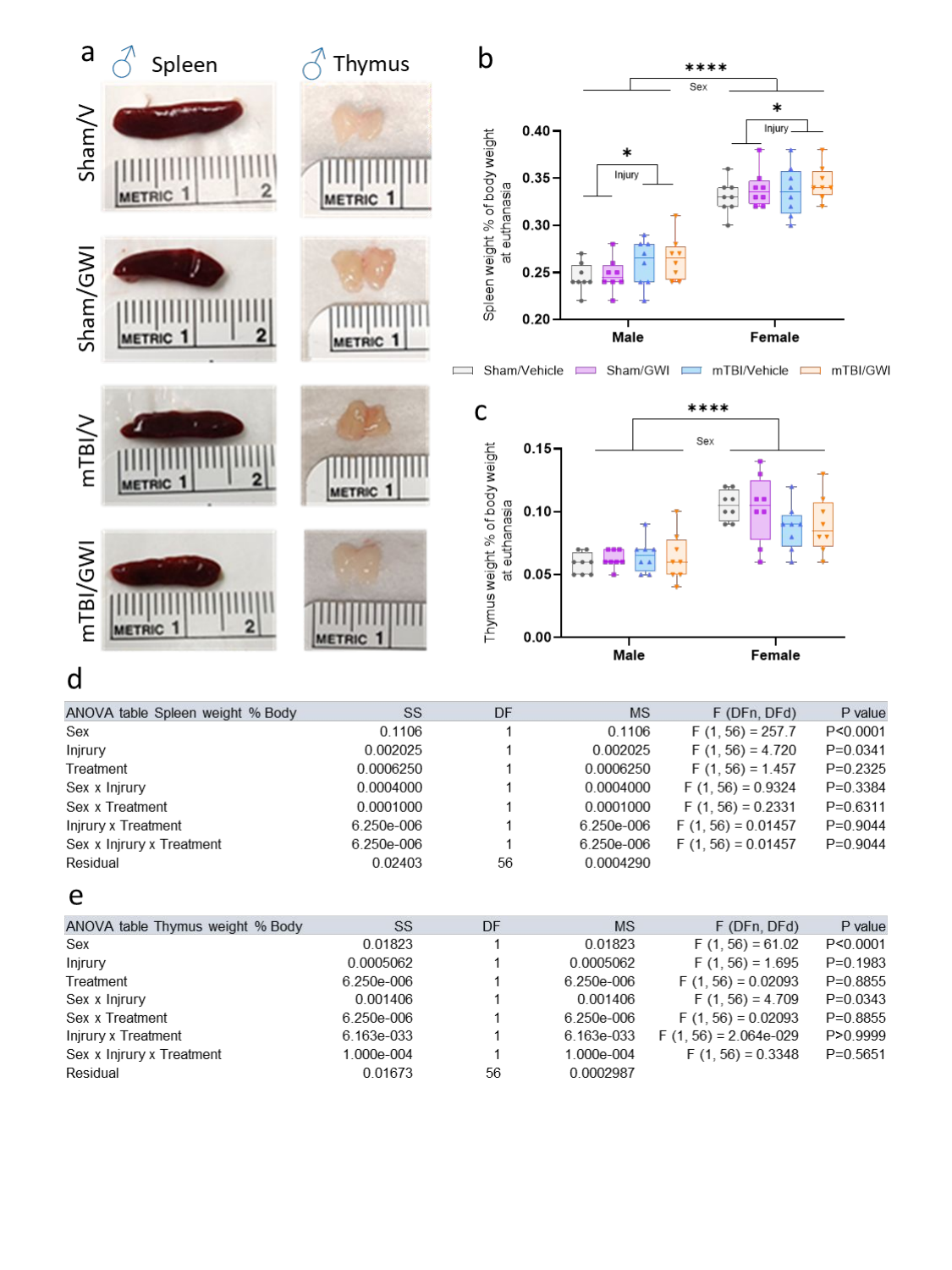

Supplement: Supplementary file 2 — Additional file 2: Figure S2. Spleen and Thymus weight in percentage of body weight at euthanasia. (a) Representative images taken from male mice at 5 months post GW treatment are shown to illustrate the spleen and thymus size. (b, c) For both tissue, a Sex effect observed due to the lower body weight of female mice (P<0.0001, 3 Way-ANOVA). (b, d) An injury effect was also observed with the injured animals having in general a larger spleen weight compared to their body weight (P=0.0341; Three-Way-ANOVA). (d, e) Results of the 3-Way ANOVA tables for the weight at euthanasia. Data are presented as Interleaved box & whiskers plots - Min to Max, 3 Way analysis of variance. [file 40478_2022_1449_MOESM2_ESM.pptx]

## Slide 1
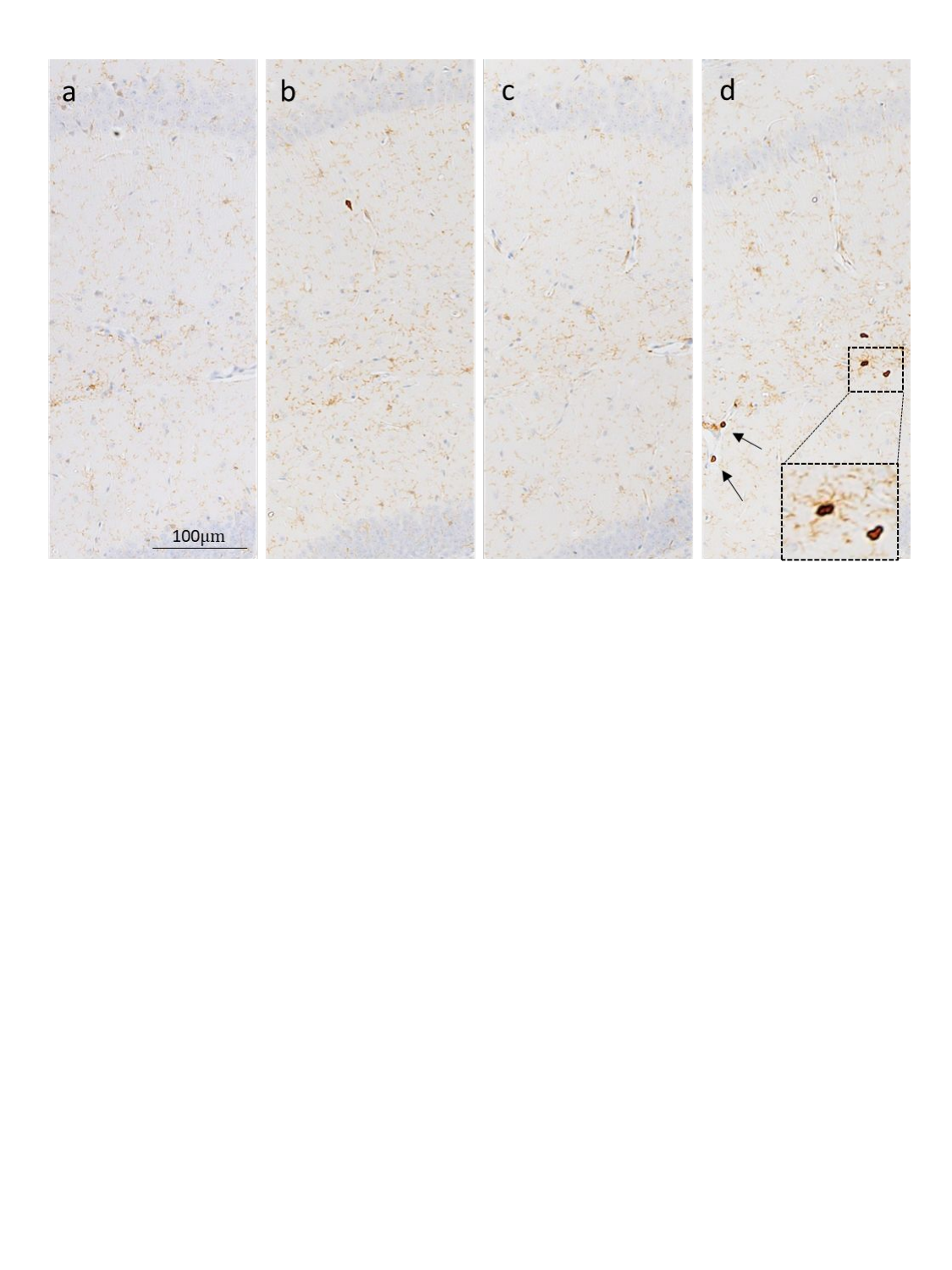

Supplement: Supplementary file 3 — Additional file 3: Figure S3. Immunostaining with cluster of differentiation receptors 45 (CD45) probing activated microglia and peripherical macrophage. at 5 months after mTBI in the hippocampus. (a–d) No changes in neurogenesis were observed at 5 months post injury or GW treatments by evaluating the % of area stained and morphology of the microglias. Qualitatively, there was a trend for an increased number of cells of with a doughnut-shaped blob (d, magnified inset), possibly as an infiltrating T cell as they were also observed within and along the blood vessels (d, arrows). [file 40478_2022_1449_MOESM3_ESM.pptx]

## Slide 1
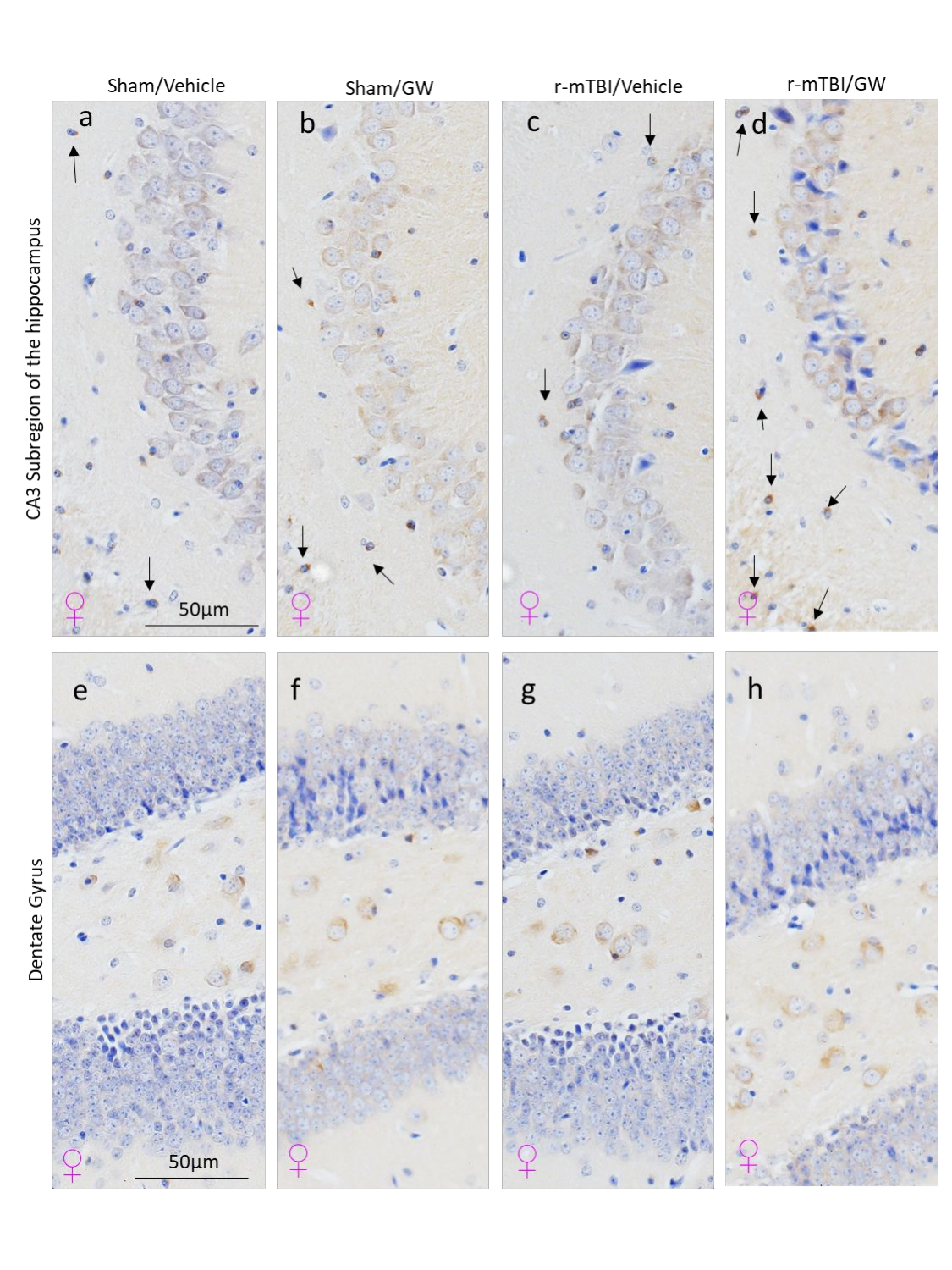

## Slide 2
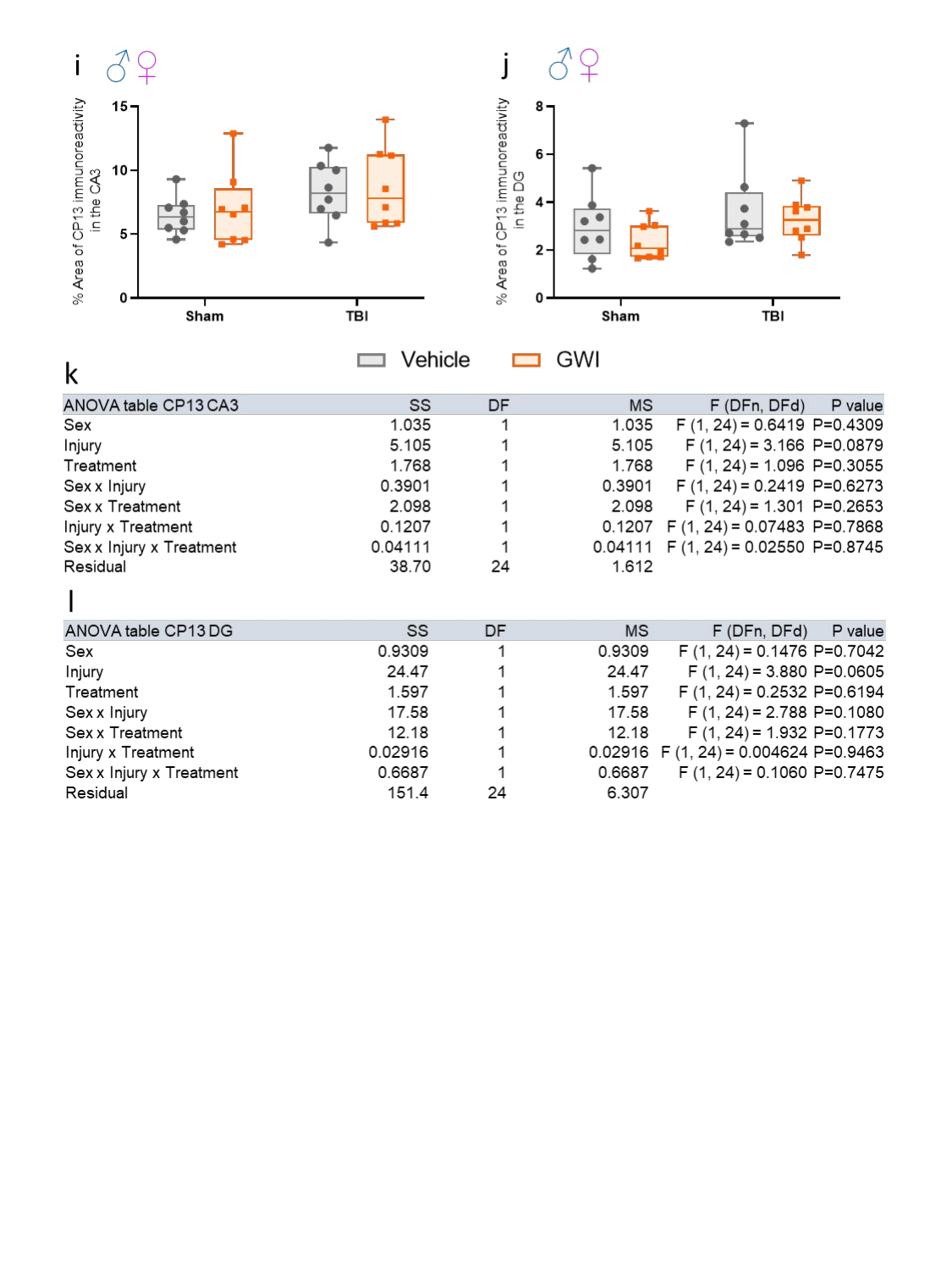

Supplement: Supplementary file 4 — Additional file 4: Figure S4. Immunohistochemical assessment of p-tau pSer-202 (CP13) in CA3 region of the hippocampus and dentate gyrus at 6 months post injury. (a–d) Gulf War treatment alone had no effect overall on the accumulation of p-Tau CP13 in the CA3 sub region of the hippocampus and in the dentate gyrus. All experimental groups exhibited physiological somatodendritic accumulation of CP13 in the hippocampus. Each symbol represents 1 mouse. (k,l) 3-Way ANOVA summary tables. Data are presented as Whiskers plots: Min to Max. Show all points; symbol represents 1 mouse n=4 per group per Sex). [file 40478_2022_1449_MOESM4_ESM.pptx]
